# Cardiac Glycoside Glucoevatromonoside Induces Cancer Type-Specific Cell Death

**DOI:** 10.3389/fphar.2018.00070

**Published:** 2018-03-01

**Authors:** Naira F. Z. Schneider, Claudia Cerella, Jin-Young Lee, Aloran Mazumder, Kyung Rok Kim, Annelise de Carvalho, Jennifer Munkert, Rodrigo M. Pádua, Wolfgang Kreis, Kyu-Won Kim, Christo Christov, Mario Dicato, Hyun-Jung Kim, Byung Woo Han, Fernão C. Braga, Cláudia M. O. Simões, Marc Diederich

**Affiliations:** ^1^Laboratorio de Virologia Applicada, Departamento de Ciências Farmacêuticas, Centro de Ciências da Saúde, Universidade Federal de Santa Catarina, Florianópolis, Brazil; ^2^Laboratoire de Biologie Moléculaire et Cellulaire du Cancer, Hôpital Kirchberg, Luxembourg, Luxembourg; ^3^Department of Pharmacy, Research Institute of Pharmaceutical Sciences, College of Pharmacy, Seoul National University, Seoul, South Korea; ^4^Department of Biology, Friedrich-Alexander Universität, Erlangen-Nürnberg, Erlangen, Germany; ^5^Departamento de Produtos Farmacêuticos, Faculdade de Farmácia, Universidade Federal de Minas Gerais, Belo Horizonte, Brazil; ^6^SNU-Harvard Neurovascular Protection Center, College of Pharmacy and Research Institute of Pharmaceutical Sciences, Seoul National University, Seoul, South Korea; ^7^Faculté de Médecine, Université de Lorraine, Nancy, France; ^8^College of Pharmacy, Chung-Ang University, Seoul, South Korea

**Keywords:** cardiac glycoside, glucoevatromonoside, apoptosis, non-canonical cell death, lung cancer

## Abstract

Cardiac glycosides (CGs) are natural compounds used traditionally to treat congestive heart diseases. Recent investigations repositioned CGs as potential anticancer agents. To discover novel cytotoxic CG scaffolds, we selected the cardenolide glucoevatromonoside (GEV) out of 46 CGs for its low nanomolar anti-lung cancer activity. GEV presented reduced toxicity toward non-cancerous cell types (lung MRC-5 and PBMC) and high-affinity binding to the Na^+^/K^+^-ATPase α subunit, assessed by computational docking. GEV-induced cell death was caspase-independent, as investigated by a multiparametric approach, and culminates in severe morphological alterations in A549 cells, monitored by transmission electron microscopy, live cell imaging and flow cytometry. This non-canonical cell death was not preceded or accompanied by exacerbation of autophagy. In the presence of GEV, markers of autophagic flux (e.g. LC3I-II conversion) were impacted, even in presence of bafilomycin A1. Cell death induction remained unaffected by calpain, cathepsin, parthanatos, or necroptosis inhibitors. Interestingly, GEV triggered caspase-dependent apoptosis in U937 acute myeloid leukemia cells, witnessing cancer-type specific cell death induction. Differential cell cycle modulation by this CG led to a G2/M arrest, cyclin B1 and p53 downregulation in A549, but not in U937 cells. We further extended the anti-cancer potential of GEV to 3D cell culture using clonogenic and spheroid formation assays and validated our findings *in vivo* by zebrafish xenografts. Altogether, GEV shows an interesting anticancer profile with the ability to exert cytotoxic effects via induction of different cell death modalities.

## Introduction

Natural products directly or indirectly provided about 50% of all clinically approved anticancer drugs between 1941 and 2014 (Newman and Cragg, 2016) and are considered as an important source of bioactive scaffolds in research and development. Cardenolides belong to the group of cardiac glycosides (CG) and attracted much interest in preclinical anticancer research. Cardiac glycosides like digoxin or digitoxin are clinically used for the treatment of heart failure and atrial arrhythmia. More recently, antiviral and anticancer activities were described (Cerella et al., 2013; Slingerland et al., 2013).

As anticancer agents, CGs triggered different cell death mechanisms including the intrinsic or the extrinsic apoptosis (Juncker et al., 2011; Cerella et al., 2015). Furthermore, CG-induced autophagic cell death was described in breast (Farah et al., 2016), ovarian (Hsu et al., 2016), colorectal (Kang et al., 2016), nerve system (Radogna et al., 2016), or lung cancer cells (Wang et al., 2012). More recently, the ability of CGs to trigger anoikis (Pongrakhananon et al., 2014) and immunogenic cell death (Menger et al., 2012, 2013; Diederich et al., 2017) were reported.

The ability of CGs to trigger non-canonical cell death modalities constitutes an advantage, especially for cancer types that developed intrinsic resistance against apoptotic cell death when treated with a broad range of chemotherapeutic agents (Diederich and Cerella, 2016; Diederich et al., 2017). Also, the capacity of CGs to exert cell-type specific anti-cancer effects will allow personalized treatments against selected cancer subtypes (Diederich et al., 2017). Considering the increased interest in non-canonical cell death modalities, new compounds presenting alternative mechanisms of cell death induction are essential and the aim of this report.

Based on a preliminary screening of 46 CGs, we focused our study on glucoevatromonoside (GEV), a cardenolide isolated from a Brazilian cultivar of *Digitalis lanata* (Castro Braga et al., 1996). In this study, we initially focused on lung cancer as one of the most common form of cancer worldwide with a poor 5-year survival rate (±25%), despite the recent implementation of targeted therapies, thus yet clearly needing new treatment avenues to be discovered. We investigated the effect of GEV on a panel of lung cancer cell lines and selected A549 (Schneider et al., 2018) as a cell type representing non-small cell lung adenocarcinoma, the most frequent histological form of lung cancer in both smokers and non-smokers. In order to provide a proof of concept of the activity of GEV, we generalized our findings on a panel of cancer cell models from different tissues, including examples of other solid and hematological forms. GEV exhibits a significant cytostatic and cytotoxic effect at nanomolar levels in adherent and non-adherent cancer cell types, without affecting healthy cell models. Our results demonstrate the capacity of GEV to activate caspase-independent cell death in the lung cancer model, validated by 2D and 3D cell culture, spheroid and colony formation assays as well as by *in vivo* zebrafish xenografts. Furthermore, here we extended our mechanistic studies to an example of hematological cancer by selecting U937 cells, which exhibit a similar susceptibility to GEV compared to A549 cells to be within a comparable concentration range for the induction of cell death modalities. Our results show in this instance the induction of a caspase-dependent apoptosis, indicating a cancer cell type-specific induction of different modalities of cell death induced by GEV.

## Materials and methods

### Cardenolides and chemicals

The origin of all tested cardenolides is indicated in Supplementary Table 1. Compounds were dissolved in dimethyl sulfoxide (DMSO) (Merck, Darmstadt, Germany). Paclitaxel was from Sigma-Aldrich (St. Louis, USA). Etoposide, 3-aminobenzamide (3-ABA), cathepsin L inhibitor, and bafilomycin A1 were from Sigma-Aldrich (Bornem, Belgium). z-VAD-FMK (z-VAD), necrostatin (Nec)-1, and calpain inhibitor PD150606 were from Calbiochem (Leuven, Belgium). Cathepsin B inhibitor was from Cell Signaling Technology (Bioke, Leiden, The Netherlands). Mammalian Target of Rapamycin (mTOR) inhibitor PP242 (Torkinib) was from Sigma-Aldrich.

### Cells

Human non-small cell lung cancer (NSCLC) A549 cells (ATCC, Manassas, USA) and normal fetal lung fibroblast cells (MRC-5, ECACC, Salisbury, UK) were grown in Dulbecco's Modified Eagle's Medium (DMEM; Gibco® Carlsbad, CA, USA) supplemented with 10% (v/v) fetal bovine serum (FBS; Gibco®). MRC-5 cells were complemented with 2 mM glutamine (Cultilab, Campinas, São Paulo, BR) and 1% non-essential amino acids (Gibco®). NSCLC cell lines H1573, H1975, H1437, and H1299 were from ATCC (LGC Standards, Molsheim, France). HT-29 (human colon adenocarcinoma), SK-N-AS and SH-SY5Y (human neuroblastoma), K562 (chronic myelogenous leukemia), U937 (acute myeloid leukemia), Jurkat (T-cell leukemia), and Raji (Burkitt's Lymphoma) cells were from DSMZ (Braunschweig, Germany); cells were cultured in RPMI medium (Lonza, Verviers, BE) supplemented with 10% (v/v) fetal calf serum (FCS) (Lonza) and 1% (v/v) antibiotic-antimycotic (penicillin, streptomycin, and amphotericin B) (BioWhittaker, Verviers, Belgium). Peripheral blood mononuclear cells (PBMCs) were purified using Ficoll-Hypaque (GE Healthcare, Roosendaal, The Netherlands). PBMCs were isolated by density gradient centrifugation from freshly collected buffy coats obtained from healthy adult human volunteers (Red Cross, Luxembourg, Luxembourg). All healthy volunteers gave informed written consent. After isolation, cells were washed twice in 1X PBS and adjusted to 2 × 10^6^ cells/mL in RPMI 1640 (supplemented with 1% antibiotic–antimycotic and 10% FCS (BioWhittaker, Verviers, Belgium). All cells lines were maintained at 37°C and 5% CO_2_ in a humidified atmosphere.

### Cell viability and proliferation assays

#### MTT and XTT assays

Cells were plated in 96-well culture plates at 6 × 10^4^ cells/well and after 24 h, cells were treated for 48 h with different concentrations of test cardenolides or paclitaxel, used as positive control. XTT cell proliferation assay (Roche, Basel, Switzerland) was used for comparative studies with the other cell lines selected (HT-29, SH-SY5Y, SK-N-AS, K562, Jurkat, Raji, and U937) according to the manufacturer's instructions as previously described (Czepukojc et al., 2014). The impact of the most efficient cardenolide in A549 cells (glucoevatromonoside; GEV) was tested at different concentrations after 24 and 48 h treatment. After the incubation period, the 50% inhibition concentration (IC_50_) of each compound was calculated as the concentration that inhibited cell metabolism by 50%, when compared to untreated controls.

#### Trypan blue exclusion assay

Trypan blue staining was used to evaluate the loss of plasma membrane integrity in A549 and U937 cells. Cells (2 × 10^5^/well in 6-well plates) were treated with GEV up for 48 h. Viable and non-viable cells were counted with a Cedex XS® cell counting system (Roche Innovatis, Basel, Switzerland). Data were normalized to the control and described as a percentage of Trypan blue-positive cells.

### Computational docking

Initial structures of the Na^+^/K^+^-ATPase complexed with CGs were obtained from the Protein Data Bank (PDB; PDB ID: 4HYT, 4RES, 4RET) (Laursen et al., 2013, 2015) and coordinates for GEV were generated using ChemBioDraw Ultra 14.0 (PerkinElmer, Waltham, MA, USA). After removing ligands in the original data, we performed computational docking using the Autodock Vina program (Trott and Olson, 2010) with the protein and the compound as receptor and ligand, respectively. Structural superposition of 4HYT, 4RES, and 4RET coordinates was performed using *WinCoot* (Emsley et al., 2010). Structural representation of docking results was done with PyMOL (The PyMOL Molecular Graphics System, Version 1.8 Schrödinger, LLC., New York, NY, USA).

### Flow cytometry analysis

#### Monocytes and lymphocytes

PBMCs were isolated and maintained as described above (Juncker et al., 2011; Czepukojc et al., 2014). After 24 h, PBMCs were treated with GEV (10–1000 nM) for 24 and 48 h and the impact on cell viability was evaluated as explained above. The experiments were performed on mixed mononuclear cells. To further identify a differential effect of GEV on lymphocytic vs. monocytic populations, fluorescence-activated cell sorting (FACSCalibur™, Becton Dickinson GmbH, Heidelberg, Germany) and a biparametric analysis forward-scattered light (FSC) vs. side-scattered light (SSC) was performed to quantify populations of monocytes and lymphocytes (Cerella et al., 2009), by using *FlowJo*® software (Tree Star, Inc.). Analysis of monocytic marker expression confirmed the quality of the monocytic sub-population (not shown). Ten thousand events/sample were recorded.

#### Cell cycle analysis

A549 and U937 cells were treated with different concentrations of GEV, at various time points. Cells were then washed twice with phosphate-buffered saline (PBS, pH 7.4), centrifuged at 500 × *g* for 5 min, and fixed with 70% ice-cold ethanol at 4°C for at least 30 min. After fixation, cells were treated with RNase (100 μg/ml), and DNA was stained with one μg/mL propidium iodide (PI) (Sigma-Aldrich) for 20 min at room temperature. Ten thousand events/sample were recorded. The distribution of cell subpopulations in each cell-cycle phase was determined using *FlowJo*® software.

### Analysis of cell death

#### Analysis of nuclear morphology

Cell death was measured in A549 and U937 cells by microscopic observation of nuclear morphology upon staining with the DNA-specific dye Hoechst (1 μg/ml Hoechst 33342; Sigma-Aldrich) as previously described (Juncker et al., 2011). Cells were treated with the indicated concentrations of GEV for 24 and 48 h, and incubated with Hoechst for 15 min at 37°C; the nuclear morphology was analyzed by fluorescence microscopy (Leica-DM IRB, Lecuit, Luxembourg, Luxembourg). Percentages of cells with fragmented or shrunken nuclei were estimated as described (Cerella et al., 2009, 2011).

#### Analysis of cellular morphology by transmission electron microscopy

A549 cells were treated with GEV with and without bafilomycin A1 (10 nM). After treatment, samples were prepared as previously described (Schnekenburger et al., 2011). Ultrathin sections were obtained with a Reichert-Jung Ultracut S microtome (Vienna, Austria). Sections were stained with uranyl acetate and lead citrate and examined with a CM12 transmission electron microscope (Philips, Eindhoven, The Netherlands).

#### Analysis of caspase activation

The activity of caspases−3/7 was measured using the Caspase-Glo® 3/7 kit from Promega (Leiden, The Netherlands). Briefly, cells were seeded (6 × 10^4^ cells/well) in a 96-well plate for 24 h and then treated with indicated concentrations of GEV for 12, 24, 36, and 48 h. The assay was performed according to the manufacturer's instructions, and luminescence was measured using a 96-well plate Orion Microplate Luminometer (Berthold, Pforzheim, Germany). Data were normalized to luminescence of untreated cells. Etoposide (VP16, 50 μM) was used as a positive control. A549 and U937 cells were pre-treated for one h with z-VAD (50 μM), then treated with either GEV or DMSO and analyzed by different approaches. Cell proliferation was monitored in real-time using an Incucyte™ Live-Cell Imaging System (Essen BioScience, Hertfordshire, United Kingdom).

#### Analysis of phosphatidylserine exposure

To determine apoptosis by FACS, A549 cells were assayed for phosphatidylserine exposure, by using the annexin V-Fluorescein isothiocyanate (FITC) Apoptosis Detection Kit I (Becton Dickinson Biosciences, Erembodegem, Belgium) per the manufacturer's instructions. A549 cells were treated with GEV and co-treated with cathepsin inhibitors B, D, and L. Stained samples were analyzed by FACS (FACSCalibur™, Becton Dickinson, San Jose, CA, USA). The distribution of cell populations was determined using FlowJo® software.

#### Analysis of other types of cell death

A549 cells (2 × 10^5^ cells/mL) were pre-treated for 1 h with the receptor-interacting protein kinase (RIPK)-1 (necroptosis) inhibitor necrostatin-1 (160 μM), the poly(ADP-ribose) polymerase (PARP)-1 inhibitor 3-aminobenzamide (3-ABA; 5 mM), the calpain inhibitor PD 150606 (25 μM), and then treated with either GEV (50 nM) or DMSO. Untreated (DMSO) and treated cells were maintained for 72 h in an incubator (37°C and 5% of CO_2_) coupled with IncuCyte™ advice imaging system (Essen Bioscience Inc., Hertfordshire, UK). Three brightness filter images were collected every three h up to 72 h. The IncuCyte™ software was used to calculate the proliferation mean from nine non-overlapping brightness contrast pictures of each well tested.

### A549 spheroid formation

Spheroids were generated by the hanging drop method. 3 × 10^3^ A549 cells were seeded in 25 μL drops of culture media on covers of Petri dishes filled with 15 mL PBS. After incubation for 96 h with GEV (0, 1, 10, 50, 100 nM), spheroids were recovered in a 6-well plate in 4 mL culture media. Images were taken (Nikon) and further analyzed by ImageJ software (http://rsb.info.nih.gov/ij/docs/index.html). 3D picture analysis and quantification were carried out using ReViSP software (https://sourceforge.net/projects/revisp/).

### Colony formation assay

A549 cells (10^3^ cells/ml) were seeded with 0, 10, 50, or 100 nM GEV and then grown in semi-solid methylcellulose medium (Methocult H4230, StemCell Technologies Inc., Vancouver, Canada) supplemented with 10% FBS. Colonies were detected after 10 days of culture by adding 1 mg/ml of 3-(4,5-dimethylthiazol-2-yl)-2,5-diphenyltetrazoliumbromide (MTT) reagent (Sigma) and were scored by ImageJ software (U.S. National Institute of Health, Bethesda, MD, USA).

### Zebrafish xenografts

Wild-type zebrafish (*Danio rerio*) were obtained from the Zebrafish International Resource Center (ZIRC, University of Oregon, OR), maintained per SNU guidelines at 28.5°C with 10 h dark/14 h light cycles. Viability and abnormal development were assessed under light microscopy (Carl Zeiss Stereo Microscope DV4, Seoul, Korea). Pictures were taken by fixing zebrafish embryos onto a glass slide with 3% methyl-cellulose (Sigma-Aldrich). For cancer xenograft assays, after mating, fertilized eggs were incubated in Danieau's solution with 0.003% of phenylthiourea (PTU) at 28.5°C for 48 h. Micropipettes for injection and anesthesia were generated from a 1.0 mm glass capillary (World Precision Instruments, FL, USA) by using a micropipette puller (Shutter Instrument, USA). 48 h post fertilization (HPF), zebrafish were anesthetized with 0.02% tricaine (Sigma, MO) and immobilized on an agar plate. A549 cells were treated for 30 h with GEV (0, 10, 50, 100 nM) and stained last 2 h of incubation with 4 μM Cell tracker CM-Dil dye (Invitrogen). 200 A549 cells were injected into the yolk sac (PV820 microinjector, World Precision Instruments, FL, USA). Subsequently, zebrafish were incubated in 96-well plates containing Danieau's solution with 0.003% phenylthiourea (PTU) at 28.5°C for 72 h. Fish were then immobilized in a drop of 3% methylcellulose in Danieau's solution on a glass slide. Pictures were taken by fluorescence microscopy (Leica DE/DM 5000B). Area of fluorescent tumors was quantified by ImageJ software.

### Western blot analysis

A549 and U937 cells (2 × 10^5^ in six-well plates) were treated with GEV and lysed with M-PER® (Mammalian Protein Extraction Reagent; Pierce, Erembodegem, Belgium) lysis buffer. After centrifugation at 10,000 × *g* for 15 min, supernatants were collected and total proteins (20–40 μg) were separated by size using sodium dodecyl sulfate—polyacrylamide gel electrophoresis (SDS-PAGE), transferred to polyvinylidene difluoride membranes (PVDF) (Amersham Hybond P, GE Healthcare, Buckinghamshire, UK) and blocked with 5% non-fat milk in PBS/Tween 20 for 1 h. Equal loading of samples was controlled using β-actin. Blots were incubated with primary antibodies: cyclin B1 (1:1000, Merck Millipore, Overijs, Belgium), p53 (1:1000, Santa Cruz Biotechnology, Boechout, Belgium), caspase-3 (1/1000, Santa Cruz Biotechnology 31A1067), p62 (1:1000, Cell Signaling Technology), beclin-1 (1:1000, Sigma-Aldrich), LC3II (1:1000, Sigma-Aldrich), and β-actin (1:10000, Sigma-Aldrich). All antibodies were diluted in a PBS-Tween 20 solution containing 5% of bovine serum albumin (BSA) or 5% of non-fat milk. After overnight incubation with primary antibodies, membranes were washed with PBS-Tween, followed by incubation for 1 h at room temperature with the corresponding secondary horseradish peroxidase-conjugated antibodies from Santa Cruz Biotechnology. After washing three times with PBS/Tween 20, specific immunoreactive proteins were visualized by ImageQuant LAS 4000® mini system (GE Healthcare) using a chemiluminescent detection reagent kit (Amersham ECL Prime, GE Healthcare).

### Statistical analysis

Results were expressed as mean ± SD of three independent experiments. Statistical analyses were performed by one-way ANOVA followed by appropriate *post-hoc* tests. A value of *p* < 0.05 was considered statistically significant. Graphs were performed with GraphPad Prism software 7 version (GraphPad, La Jolla, CA, USA).

## Results

### Screening of 46 cardenolides identified glucoevatromonoside as a potent anticancer cardiac glycoside candidate

**Figure 1:**
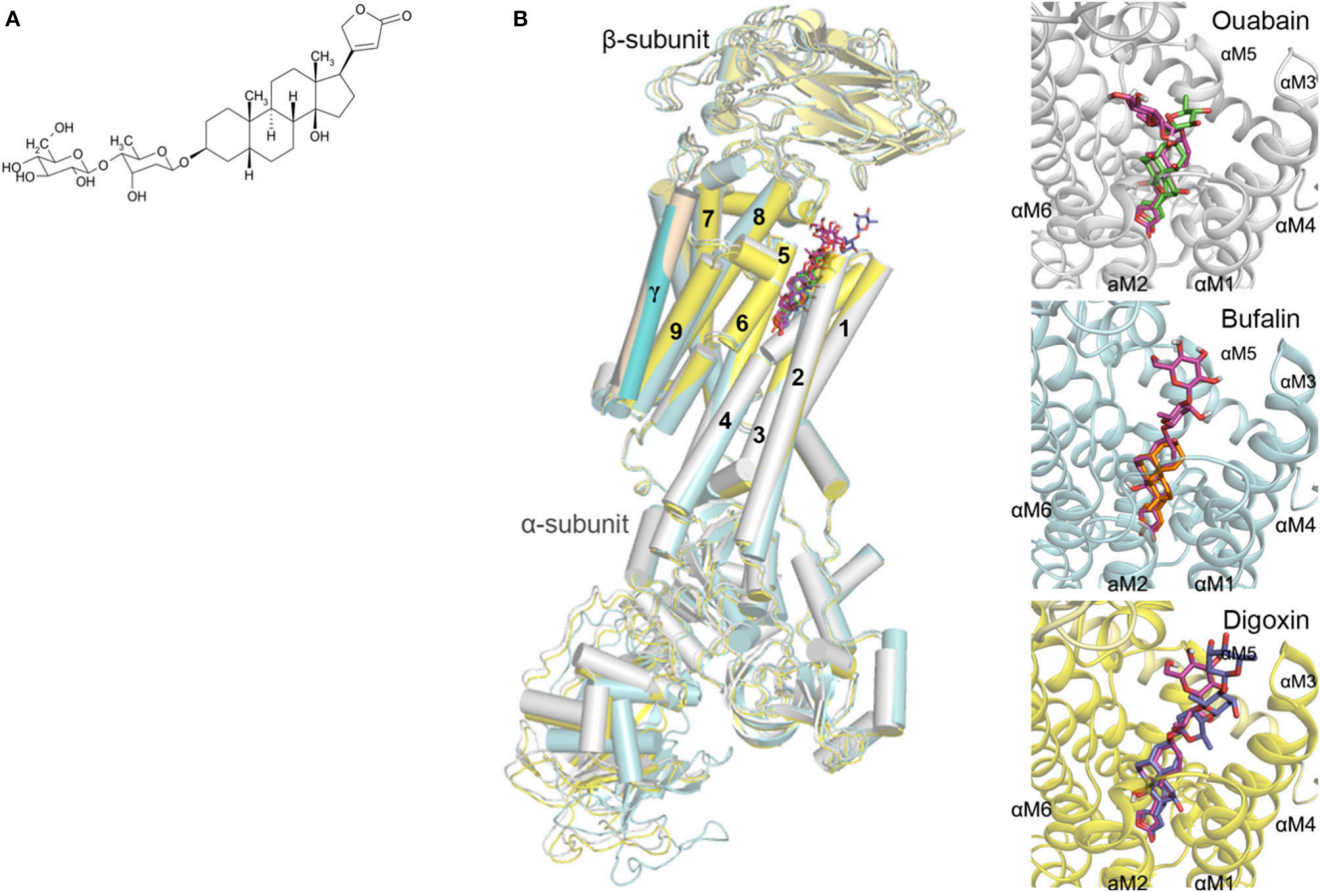
**(A)** Chemical structure of GEV; **(B)** Docking orientation of GEV in the crystal structures of the Na^+^/K^+^-ATPase (PDB ID: 4HYT, 4RES, 4RET) (Laursen et al., 2013, 2015). The Na^+^/K^+^-ATPase protein complexed with ouabain, bufalin, and digoxin is depicted as a cartoon colored in white, cyan, and yellow, respectively. The predicted docking modes of GEV are shown as stick models colored in magenta, and the coordinates of ouabain, bufalin, and digoxin are represented by green, orange, and blue sticks, respectively. The hydrogen and oxygen atoms are colored in white and red, respectively.

We evaluated the anticancer potential of a panel of 46 natural or hemisynthetic cardenolides on A549 cells (Supplementary Table 1). The most cytotoxic cardenolide was glucoevatromonoside (GEV, Figure 1A) which showed 14-fold greater potency compared to paclitaxel used as a positive control (IC_50_ = 19.3±4.5 nM vs. 260.5±70.8 nM).

### Evaluation of the cytotoxic potential of glucoevatromonoside in a panel of cancerous vs. non-cancerous human cells

We then further evaluated the anti-cancer potential and selectivity of GEV. We generalized our findings with additional lung cancer cell lines and other types of solid tumor compared to non-adherent cancer models (Table 1). Our results show that GEV was cytotoxic at nanomolar concentrations and presented selectivity for lung cancer cell models (H1299 = H1573 = H1975 = A549 = U937 > SH-SY5Y > JURKAT > K562 > RAJI > SK-N-AS > H1437 >HT-29 cells).

**Table 1:** Effect of glucoevatromonoside on cancer and non-cancer cell growth.

**Cell type**	**Cell line**	**IC50 (nM)**
Non-small cell lung cancer (carcinoma)	H1299	15.3 ± 1.5
Lung adenocarcinoma (from a metastatic site)	H1573	17.4 ± 3.3
Epithelial lung carcinoma	A549	19.3 ± 4.5
Non-small cell lung cancer (adenocarcinoma)	H1975	19.5 ± 5.9
Non-small cell lung cancer (adenocarcinoma)	H1437	63.0 ± 24.3
Neuroblastoma (neuronal origin)	SH-SY5Y	32.9 ± 16.0
Neuroblastoma (stromal origin)	SK-N-AS	56.5 ± 7.0
Colorectal adenocarcinoma	HT-29	120.8 ± 7.7
Acute myeloid leukemia	U937	19.0 ± 5.9
T lymphoblastoid cells	Jurkat	35.2 ± 9.8
Chronic myelogenous leukemia	K562	39.1 ± 3.3
Burkitt lymphoma	RAJI	53.0 ± 4.2
Normal fibroblast-like lung cells	MRC-5	161.9 ± 13.7
Normal peripheral blood mononuclear cells	PBMC	869.3 ± 178.6

*The IC_50_ value was defined as the compound concentration needed to inhibit 50% of the cell growth compared to untreated controls. Data represent the mean ± SD of three independent experiments*.

To assess for differential toxicity, we used two non-cancer cell models, lung MRC-5 cells and PBMCs from healthy donors. After 48 h of treatment, GEV was 45-fold and 8.5-fold more selective to A549 compared to PBMCs and MRC-5 cells, respectively (Table 1). The effect of GEV on sub-populations of PBMCs was also investigated (Supplementary Figures 1A,B). Results showed that GEV did not affect the lymphocytic population at any concentration (Supplementary Figure 1B). The population of monocytes was affected by GEV after 48h of treatment only at concentrations beyond the range of this investigation (1000 nM) (Supplementary Figure 1A). Considering the differential toxicity and selectivity of GEV toward lung cancer cell models, we selected the lung A549 cells for *in vitro* and *in vivo* investigations. Furthermore, we compared results to the acute myeloid leukemia cell line U937, which exhibits a similar susceptibility to GEV as A549 cells.

### Target validation

We previously documented the inhibitory potential of GEV on Na^+^/K^+^-ATPase activity (Bertol et al., 2011) by an *in vitro* assay (Hu et al., 2009). To validate the interaction mode of GEV and its putative pharmacological target, the Na+/K+ ATPase, we implemented docking simulations using Autodock Vina software (Trott and Olson, 2010) (Figure 1B). In the absence of structural data for the human Na^+^/K^+^-ATPase protein, we instead used the structure of the Na^+^/K^+^-ATPase of *Sus scrofa* complexed with three different CGs, ouabain, bufalin, and digoxin (Laursen et al., 2013, 2015) as previously published (Zeino et al., 2015). Sequence identities of α1, β1, and γ-subunits of the Na^+^/K^+^-ATPase between human and pig are 98.1, 92.4, and 80.3%, respectively (Sievers et al., 2011). Before predicting the affinity between GEV and Na^+^/K^+^-ATPase, we performed a control docking experiment with the complexed CGs as ligands, after the CGs were removed from the respective complex structures. The predicted affinity energies of GEV in three different complex structures of ouabain, bufalin, and digoxin were comparable to those calculated from the original complex structures; −11.3 kcal/mol, −11.0 kcal/mol, and −11.4 kcal/mol, against −11.1 kcal/mol, −11.1 kcal/mol, and −16.3 kcal/mol, respectively (RMSD distances between docking results and crystal structures of ouabain, bufalin, and digoxin were 0.046 Å, 0.042 Å, and 0.001 Å, respectively) (Figure 1B).

In the docking analyses, GEV was predicted to be located in the cavity of the extracellular ion pathway in the α subunit of the Na^+^/K^+^-ATPase where CGs are bound. Along with three CGs in crystal structures, the five-membered lactone ring of GEV was located in the deeper site of the cavity and the steroid core was stabilized by interacting with the transmembrane helices αM1-6. As shown in Figure 1B, the predicted docking modes of the steroid core are similar to the conformation of CGs in the crystal structure complex, as the α-surface of the steroid cores were facing to the hydrophobic side chains of αM4-6 and the β-surface of the steroid cores were located near the polar side chains of αM1-2. Predicted docking orientation of the disaccharide of GEV was different from that of ouabain as well as from those of bufalin and digoxin. In the case of a docking mode based on the ouabain complex structure, the disaccharide was stretched to the αM1-2 loop. On the other hand, based on the bufalin and digoxin complex structure the disaccharide was in proximity to the αM7-8 loop. When we compared the crystal structure in complex with ouabain and with digoxin, the αM7-8 loop of digoxin complexed structure was located closer to the ligand than that of the ouabain complexed structure. Hence, the docking program calculated that GEV best interacts with the digoxin complexed structure through interaction with the αM7-8 loop. In the case of the bufalin complexed structure, the pore is narrower than the other structures as the αM1-2 loop is located closer to the cavity, and the six-membered lactone moiety of bufalin reaches deeper into the cavity. For these reasons, the GEV disaccharide likely interacts with the αM7-8 loop and the remaining part of the GEV molecule is located deeper in the cavity occupying the area enlarged by the bulky lactone moiety of bufalin. Altogether, based on our docking studies, we hypothesized that GEV achieves its cytotoxicity by inhibiting Na^+^/K^+^-ATPase.

### Cytostatic and cytotoxic effects of glucoevatromonoside on A549 cells

**Figure 2:**
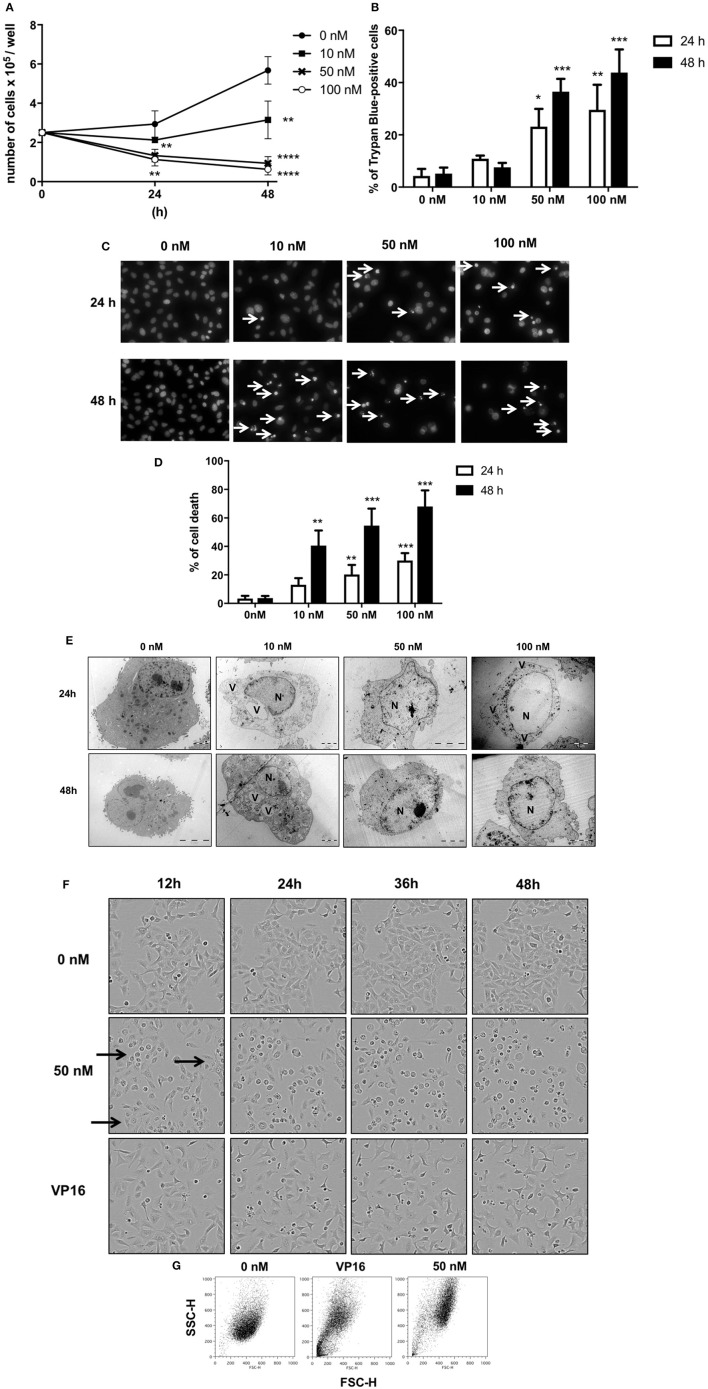
Glucoevatromonoside exerts cytostatic and cytotoxic effects on A549 cells. **(A)** GEV reduces proliferation of A549 cells in a concentration-dependent manner; **(B)** analysis of cell death by trypan blue staining after 24 and 48 h of treatment at 10, 50, and 100 nM; **(C)** Hoechst staining; and **(D)** quantification of cell fractions presenting shrinking and fragmented nuclei (white arrows) in treated cells vs. untreated cells; **(E)** Representative transmission electron microscopy of A549 cells treated with 10, 50, and 100 nM of GEV after 24 and 48 h of treatment. Images show nuclear and cytoplasmic alterations. (N) nucleus and (V) vacuoles **(F)** Representative bright filter images obtained by IncuCyte™ videomicroscopy of cells treated with GEV (50 nM) and VP16 (50 μM) after 12, 24, 36, and 48 h **(G)** Flow cytometry analysis of forward-scattered light (FSC) vs. side-scattered light (SSC) of A549 cells treated with GEV (50 nM) and VP16 (50 μM) for 48 h. Arrows represent cells with increased cell volume and granularity. Statistical analysis: ANOVA-one way; Dunnett's *post-hoc* analysis in **(C,E)**. Significance is reported as: *P < 0.05, **P < 0.01, ***P < 0.001, ****P < 0.0001 compared to controls.

GEV reduced the proliferation of A549 cells in a concentration and time-dependent manner (Figures 2A,B) concomitant with the accumulation of cell death after treatment with 50 and 100 nM GEV (24 h: 21% and 36%; 48 h: 40% and 53%). Observation by fluorescence microscopy of Hoechst-stained nuclei confirmed the progressive accumulation of cells displaying pyknotic nuclei, besides a minor additional fraction with nuclear fragmentation (Figures 2C,D). Analysis by transmission electron microscopy (TEM) further validated severe ultrastructural alterations of cytoplasm and nucleus in GEV-treated A549 cells. After 24 and 48 h, cells committed to death showed a disruption of the cytoplasmic architecture with an accumulation of large vacuoles, resembling intracellular edemas. Meanwhile, nuclei were shrunken but not fragmented, presenting locally condensed chromatin (Figure 2E). Remarkably, no cells with intermediate levels of degeneration were observed, suggesting a rapid evolution after cell death commitment. Real-time videomicroscopy of A549 cells treated with GEV at 50 nM confirmed a peculiar evolution of cellular alterations compared to etoposide (VP16), a canonical apoptosis inducer, used as a control (Figure 2F and Supplementary Videos 1–3). Whereas, VP16 sequentially triggered shrinkage, fusiform morphology, detachment, and fragmentation, GEV induced increased cell volume and granularity as quantified by flow cytometry (Figure 2G). Altogether, these observations suggested that GEV induced a differential, non-apoptotic, cell death pathway.

### Molecular pathways leading to glucoevatromonoside-induced cell death

**Figure 3:**
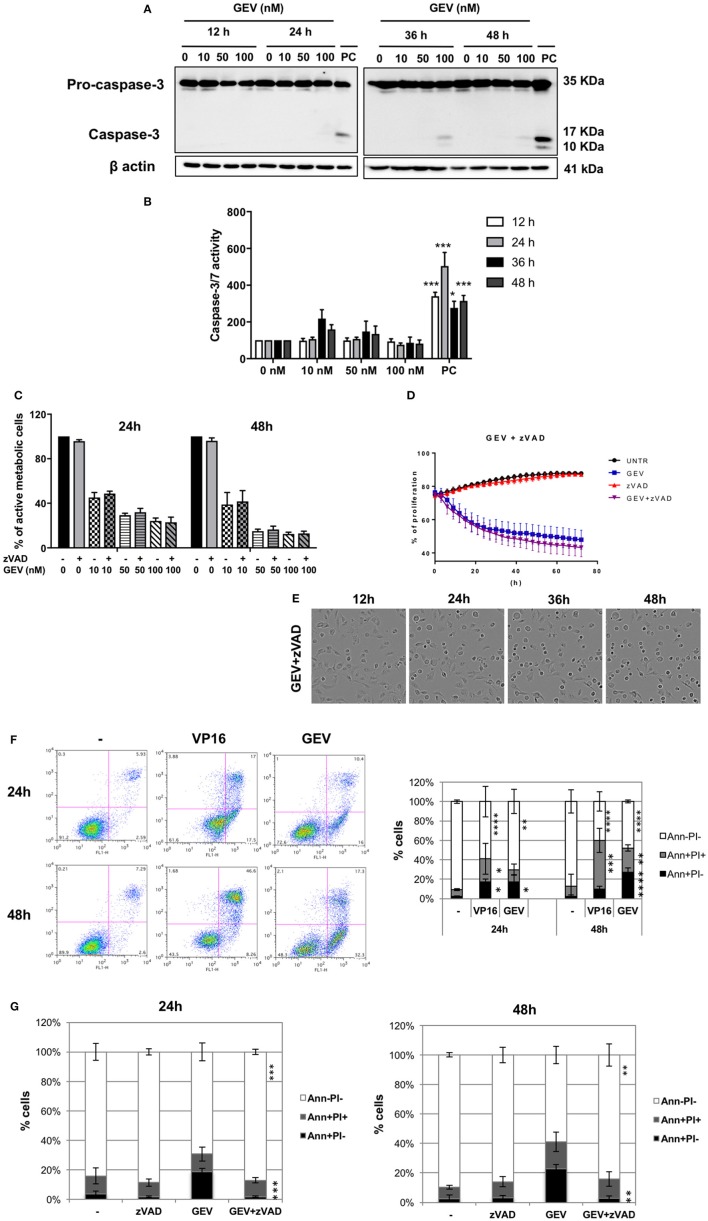
Glucoevatromonoside induces cell death with hybrid characteristics. **(A)** A549 cells were treated with GEV, extracted proteins were separated by SDS-PAGE and analyzed by Western blot for caspase-3 cleavage. As a positive control (PC), U937 cells were treated with etoposide (50 nM VP16, 3 h); **(B)** Quantification of substrate cleavage using luminescence-based assays. Cells were incubated with GEV at 10, 50, and 100 nM and the activation of caspase-3 and−7 was investigated using a luminescence assay. Caspase activity was monitored after 12, 24, 36, and 48 h. As a positive control, A549 cells were treated with etoposide (50 μM VP16). Data were normalized to untreated controls; **(C)** Percentage of cells protected by z-VAD against a GEV-induced reduction of viability. **(D)** The proliferation of A549 cells after 72 h of GEV treatment with and without z-VAD **(E)** Representative bright filter images obtained with IncuCyte™ videomicroscopy of cells treated with GEV (50 nM) and z-VAD after 12, 24, 36, and 48 h. The data represent the mean ± SD of three independent experiments. *p < 0.05 (ANOVA followed by Dunnett's test) when compared to untreated controls. **(F)** A549 cells were treated with GEV (50 nM) and VP16 (50 μM) for 24 and 48 h, stained with annexin-V-FITC and PI and analyzed by flow cytometry. In each panel, the lower left quadrant shows cells negative for both annexin-V-FITC and PI (Ann-PI-), lower right quadrant shows annexin-V positive cells which are in the early stage of apoptosis (Ann+PI-), upper left quadrant shows PI positive cells, and the upper right quadrant shows both annexin V and PI positive (Ann+PI+) cells **(G)** Percentage of Ann+PI-, Ann+PI+, and Ann-PI- after 24 and 48 h of GEV treatment. Data shown represent the mean ± SD of three independent experiments [ANOVA-two way; Dunnett's **(E)** or Sidak **(F)** *post-hoc* analysis]. *P < 0.05, **P < 0.01, ***P < 0.001, ****P < 0.0001 compared to controls.

GEV treatment did not result in a significant level of caspase-3 cleavage or activity even at 100 nM after 48 h (Figures 3A,B), in contrast to VP16. Moreover, the pan-caspase inhibitor z-VAD did not prevent GEV-induced cell death (Figures 3C,D) or morphological alterations of A549 cells (Figure 3E and Supplementary Videos 1–5).

**Figure 4:**
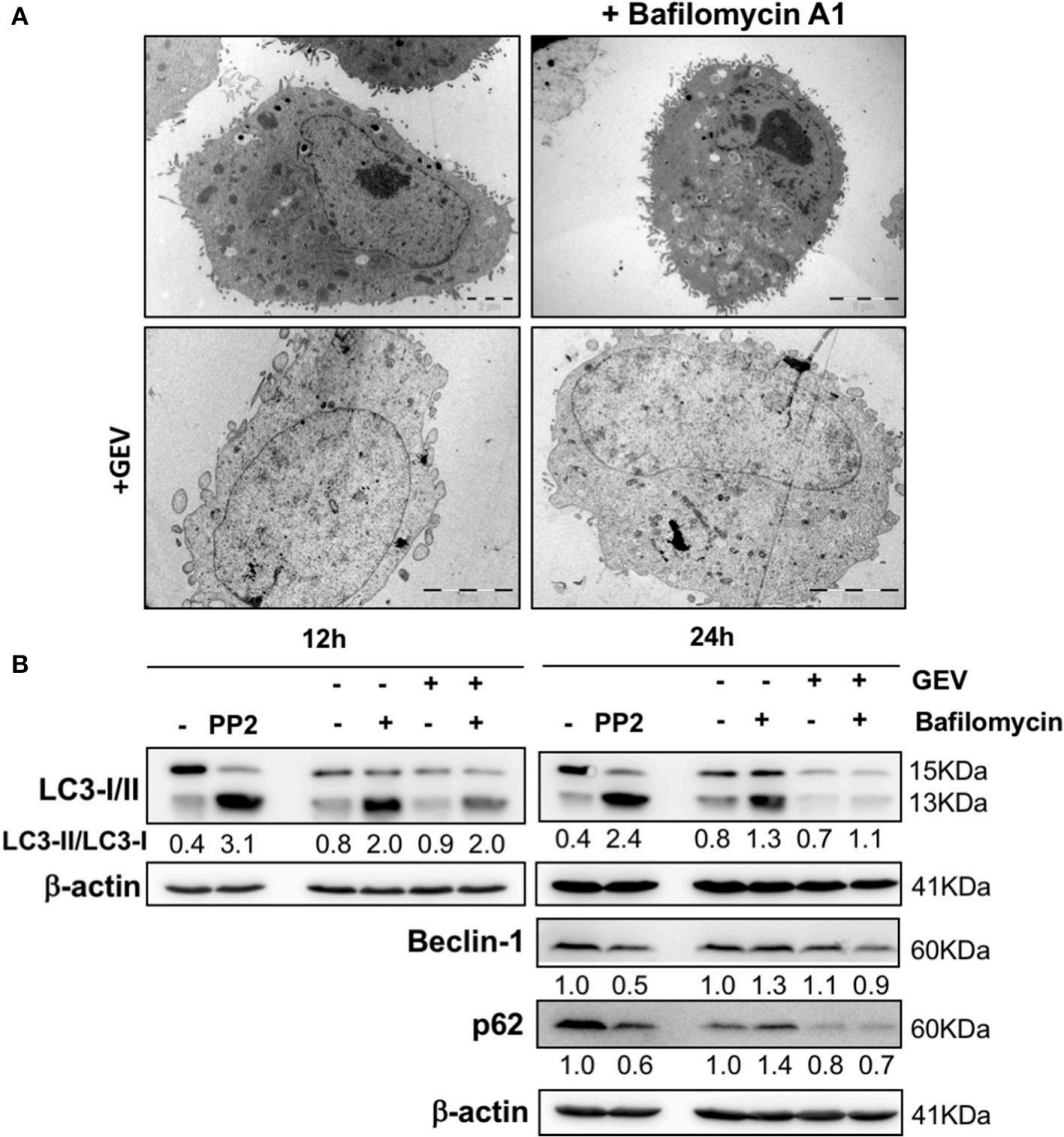
Effect of glucoevatromonoside on autophagic flux. **(A)** Representative transmission electron microscopy of A549 cells treated with GEV (50 nM) with and without bafilomycin A1 (10 nM) after 12 and 24 h of treatment. **(B)** Western blot analysis of LC3-II, p62 and beclin-1 protein levels in A549 cells treated with GEV (50 nM) and bafilomycin A1. Mammalian Target of Rapamycin (mTOR) inhibitor PP242 (Torkinib) was used as a positive control. ™-actin was used as a loading control. The blots shown are representative of three independent experiments. Expression levels of proteins were quantified by using ImageJ software (National Institutes of Health, Bethesda, USA). Numbers below western blot signal represent quantification of LC3II/LC3-I proteins ratio using β-actin as control for sample input.

Considering the capacity of CGs to trigger autophagy and to verify autophagy involvement in GEV-treated cells, we monitored cell morphology by TEM (Figure 4A), together with the assessment of LC3I-II conversion, p62 and beclin-1 expression levels at earlier time points and different concentrations (Figure 4B and Supplementary Figure 2) in presence/absence of bafilomycin A1. All results excluded the induction of parossistic autophagy by GEV. Rather, the robust autophagic flux of A549 cells was abrogated by this treatment (Figure 4B and Supplementary Figure 2).

To assess for specific non-canonical cell death pathways potentially triggered by GEV, we treated A549 cells with poly-(ADP-ribosyl) polymerase (PARP)-1 inhibitor 3-aminobenzamide (3-ABA), the receptor-interacting protein kinase 1 (RIP-1) inhibitor necrostatin-1 as well as the calpain inhibitor PD 150606 which were unable to prevent GEV-induced reduction of A549 viability. Results were confirmed by real-time imaging (Supplementary Figure 3 and Supplementary Videos 6–11).

Of note, annexin-V-FITC/PI staining revealed an accumulation of cells exposing phosphatidylserine (PS) over the time of treatment with GEV, which could be prevented by the pan-caspase inhibitor z-VAD but not by several cathepsin inhibitors (Figures 3E,F and Supplementary Figure 4). This result confirms caspase-dependency involved in PS exposure and points at two possible alternative scenarios where apoptotic and non-apoptotic features of cell death might co-exist in different cell subpopulations or the same cell.

### Glucoevatromonoside induces apoptosis in U937 cells

**Figure 5:**
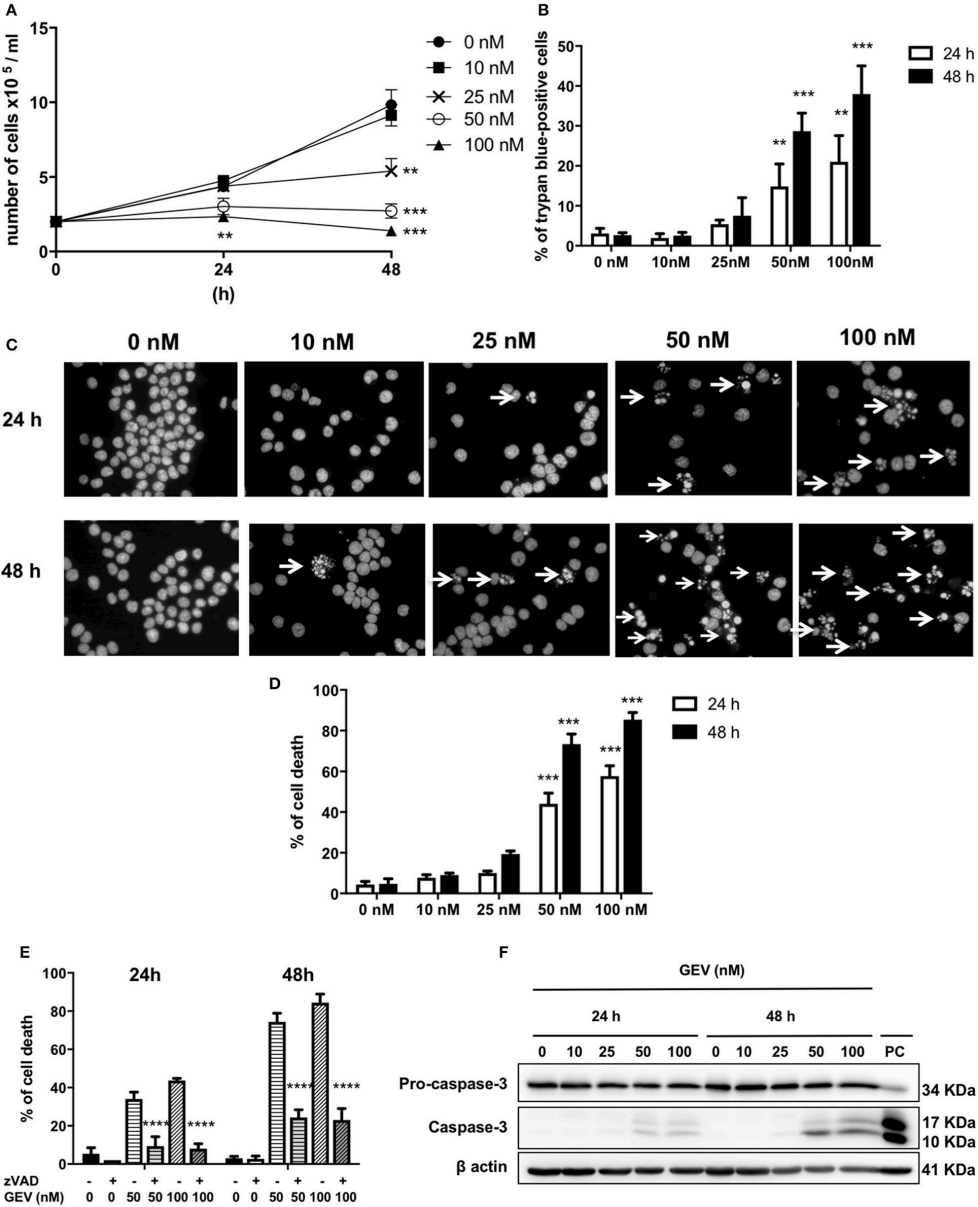
Glucoevatromonoside impairs cell growth and induces caspase-dependent apoptosis in U937. **(A)** GEV reduces proliferation of U937 cells in a concentration-dependent manner; **(B)** Analysis of cell death performed by trypan blue staining after 24 and 48 h of treatment at 10, 50, and 100 nM; **(C)** GEV induces apoptotic nuclear morphology. Arrows show fragmented nuclei; **(D)** Quantification of cell death by Hoechst staining of fragmented nuclei in treated cells vs. untreated cells; **(E)** Effect of zVAD on cell death induction by GEV at 50 and 100 nM evaluated by Hoechst staining; **(F)** U937 cells were treated with GEV, extracted proteins were separated by SDS-PAGE and analyzed by Western blot against caspase-3. Etoposide was used as positive control at 50 μM for 3 h. Data represent the mean ± SD of three independent experiments (ANOVA followed by Dunnett's test). Significance is reported as: **P < 0.01, ***P < 0.001, ****P < 0.0001 compared to controls.

Hematopoietic non-adherent U937 cells were as susceptible to GEV as the lung epithelial A549 cells (Table 1). Figure 5A shows a reduction of the proliferation of U937 cells in a concentration-dependent manner, similarly to A459 cells. This effect was accompanied by the accumulation of around 40% of Trypan blue-positive cells after 48 h of treatment with 100 nM GEV (Figure 5B). GEV-treated U937 cells undergo changes in their nuclear morphology in a concentration-dependent manner leading to apoptotic nuclear condensation and fragmentation (Figures 5C,D). GEV induced cleavage of caspase-3 after 24 and 48 h detectable from 50 nM (Figure 5F) like etoposide. Moreover, z-VAD significantly rescued over 50% of U937 cells from nuclear fragmentation (Figure 5E). Altogether, these results indicate the ability of GEV of triggering a caspase-dependent apoptosis in U937 cells.

### Glucoevatromonoside triggers differential cell cycle inhibition in A549 and U937 cells

**Figure 6:**
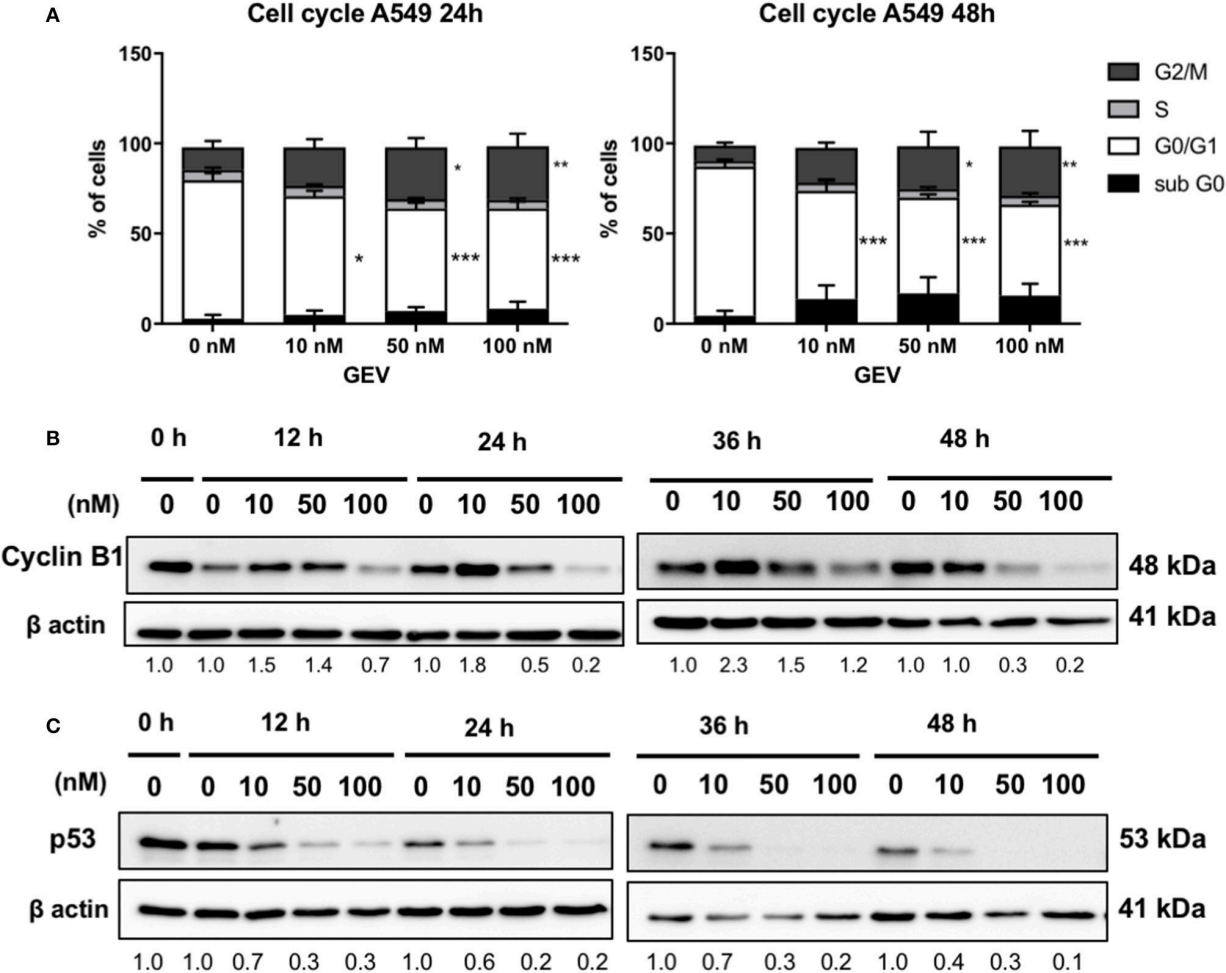
Glucoevatromonoside induces cell cycle arrest. **(A)** GEV causes accumulation of A549 cells in G2/M. Cell cycle analysis of cells treated with GEV for 24 and 48 h. Percentages of A549 cells in subG0, G0/G1, S, and G2/M are indicated. DMSO served as vehicle control. The data represent the mean ± SD of three independent experiments; *p < 0.05, **p < 0.01, and ***p < 0.001 (ANOVA followed by Dunnett's test) when compared to untreated controls; Western blot analysis of cyclin B1 **(B)** and p53 **(C)** expression in cells with and without GEV at 10, 50, and 100 nM, for 24 and 48 h. DMSO served as vehicle control. ®-actin was used as loading control. The blots shown are representative of three independent experiments. Expression levels of cyclin B1 and p53 were quantified by using ImageJ software (National Institutes of Health, Bethesda, USA). Values below western blots represent quantification of hybridization signals using β-actin for normalization. Significance is reported as: *P < 0.05, **P < 0.01, ***P < 0.001 compared to controls.

GEV induced an accumulation of approximately 30% cells in the G2/M phase after 24 and 48 h in A549 cells, accompanied by a decrease of cells in G1 phase and a small increase of cells in sub-G0/G1 phase concomitant with cyclin B1 accumulation at 10 nM followed by a reduction at 50 and 100 nM as early as 12 h. It also down-regulated p53 at all concentrations tested as earlier as 12 h (Figure 6). In U937 cells, GEV (100 nM) led to the accumulation of 32 and 81% cells in sub-G0/G1 phase after 24 and 48 h, respectively, with a corresponding decrease of cells in G1, compared to untreated controls (Supplementary Figure 5).

### Glucoevatromonoside abrogates tumor formation in a 3d culture environment and zebrafish xenografts

**Figure 7:**
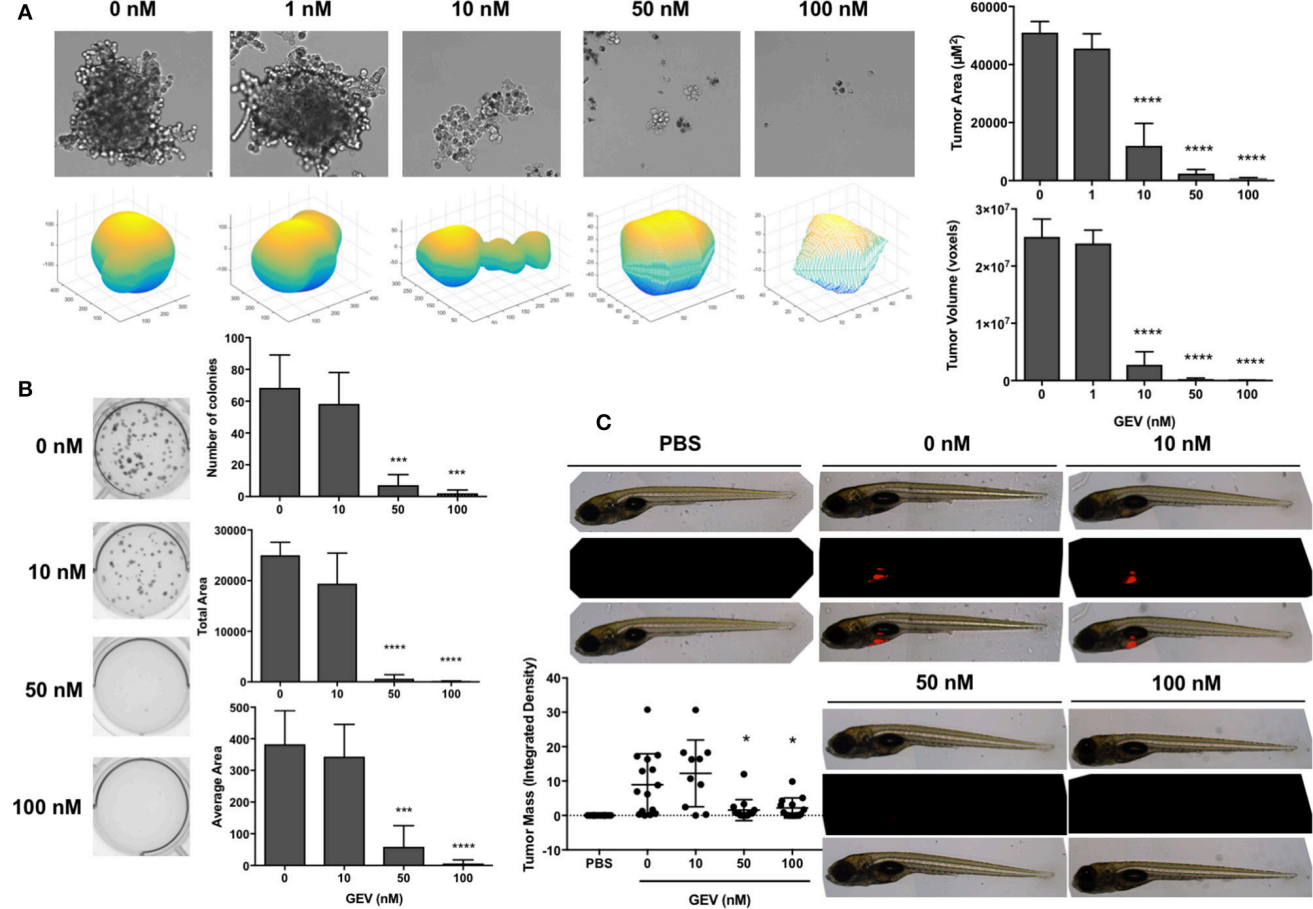
Glucoevatromonoside abrogates tumor formation in 3D culture and zebrafish xenografts. **(A)** Representative bright field images and 3D plots of spheroids generated after dose-dependent treatment of A549 by GEV (mean of three independent 4-day experiments ± SD). Quantification of tumor area and volume are shown; **(B)** A549 colony formation assays in the presence of increasing concentrations of GEV (10 days). Number of colonies, total, and average area are reported (mean of four independent experiments ± SD); **(C)** effect of GEV on tumor formation in a zebrafish A549 xenograft assay. CM-dil-stained A549 cells were treated for 30 h with 0, 10, 50, and 100 nM GEV and injected in the zebrafish yolk sac at 48 hpf. Representative images from at least 10 fish per condition. PBS injection served as a negative control. Fluorescent tumor mass was quantified and reported in the graph. Statistical analysis by ANOVA-one way; Dunnett's *post-hoc* test. Significance is reported as: *P < 0.05, ***P < 0.001, ****P < 0.0001 compared to controls.

We then confirmed the potential of GEV to impair the ability of A549 cells in *in vitro* and *in vivo* 3D tumor formation assays in the presence of increasing concentrations of the compound. GEV strongly prevented A549 spheroid formation at 10 nM and completely abrogated their development at 50 nM (Figure 7A). In addition, colony formation was completely abolished by GEV at 50 nM (Figure 7B). To extend our colony formation assays to an *in vivo* situation, we assessed the capacity of GEV to abrogate A549 tumor formation in a zebrafish xenograft model. Our results confirmed a strong abrogation of tumor formation after injection of 50 nM GEV-pretreated, fluorescently stained A549, validating our *in vitro* results (Figure 7C).

## Discussion

The potential of CGs as anticancer drugs arose from the epidemiological evidence of a lower mortality rate of patients suffering from both congestive heart failure and breast cancer under treatment with digoxin (Haux, 1999; Stenkvist, 2001). Since then, anticancer activity of CGs have been evaluated *in vitro* (Newman et al., 2007; Hundeshagen et al., 2011; Juncker et al., 2011; Elbaz et al., 2012; Wang et al., 2012; Xue et al., 2015) and *in vivo* (Svensson et al., 2005). The aqueous extract of *Nerium oleander* containing oleandrin (Anvirzel™) (Mekhail et al., 2006; Hong et al., 2014), digoxin or digitoxin were assessed in clinical trials against different types of tumors (Prassas et al., 2011). Repositioning of this class of clinically-validated drugs could allow reducing cancer drug development time and costs (Duenas-Gonzalez et al., 2008; Cragg et al., 2014).

Here GEV was the most potent of 46 CGs tested with a cytotoxic potential above clinically-used paclitaxel (Tsvetkova and Goss, 2012). To validate the putative pharmacological target of GEV, we implemented docking analyses using the Autodock Vina program (Trott and Olson, 2010). When we run the docking simulation based on crystal structures of the pig kidney Na^+^/K^+^-ATPase complexed with various CGs (Laursen et al., 2013, 2015), GEV was predicted to bind the extracellular ion pathway of Na^+^/K^+^-ATPase corresponding to the CG binding site. The predicted affinity energies of GEV bound to ouabain and bufalin complexed structures were similar to those of their original ligands, but predicted binding energy of digoxin was higher than that of GEV. When we analyzed the docking results, the disaccharide did not fully occupy the cavity formed by the α- and β-subunits of the Na^+^/K^+^-ATPase, whereas the three control CGs can fully inhibit this cavity by interacting with Gln84 in the β-subunit through hydrogen bond formation. Altogether, our results show that GEV seemed to target the Na^+^/K^+^-ATPase with high affinity similarly to the control CGs.

GEV was able to trigger caspase-dependent and -independent cell death mechanisms, depending on the cell context. Of note, GEV presented an interesting differential toxicity. Furthermore, *in vitro* 3D cell culture using clonogenic and spheroid formation assays as well as *in vivo* investigations on zebrafish xenograft validated our findings, providing a pre-clinical favorable profile for GEV as new anti-cancer agent. Overall, lung cancer cell models with different genetic backgrounds were very sensitive, encouraging further investigations on this type of cancer.

Our results are in line with previous investigations showing the ability for a same CG to induce cytotoxic effects via execution of different forms of cell death, including UNBS1450, oleandrin, and digoxin (Mijatovic et al., 2006; Newman et al., 2008; Juncker et al., 2011). UNBS1450, for example, could induce autophagy in lung, prostate, pancreatic, and brain cancer cell lines (Mijatovic et al., 2006, 2008; Kulikov et al., 2007; Newman et al., 2007; Lefranc and Kiss, 2008), while apoptosis induction was observed in melanoma and leukemia (Mathieu et al., 2009; Juncker et al., 2011). Interesting prototypical inhibitors of controlled necrosis were unable to protect cells against GEV excluding these alternative mechanisms of cell death induction. Surprisingly, GEV prompts the exposure of phosphatidylserine on the outer leaflet of the plasma membrane in A549 cells correlated to caspase involvement, shown by zVAD-sensitive PS exposure. This result might suggest an attempt by A549 cells to activate a canonical caspase-dependent cell death program, however aborted in consequence of rapid and dramatic loss of cell volume homeostasis, as witnessed by the appearance of extensive intracellular edemas and cell morphology/volume deregulation.

CGs displayed anti-proliferative activity via cell cycle inhibition (Mijatovic et al., 2012) essentially G2/M (Feng et al., 2010; Xue et al., 2015) or S phase (Xu et al., 2011) accumulation. Our results are in line with Wang and coworkers (Wang et al., 2012) investigating the effect of clinically-used digoxin and ouabain. Authors reported no significant alterations in G1 or S phases but a differential increase of G2/M arrest in A549 and H460 cells (Wang et al., 2012). In U937 cells, GEV showed essentially an increase of cells in subG0 phase in agreement with our results obtained with the hemisynthetic CG UNBS1450 in various hematopoietic cancer cells (Schumacher et al., 2011). Consequently, cyclin B1 expression was increased after GEV treatment at 10 nM followed by a decrease at highest concentrations. Elevated cyclin B1 expression levels are a common feature of various cancer models (Dutta et al., 1995; Mashal et al., 1996; Kushner et al., 1999; Yasuda et al., 2002; Grabsch et al., 2004) and associated with a poor prognosis for cancer patients (Soria et al., 2000). Cyclin B-cyclin dependent kinase (CDK) complexes are active regulators of the progression of the cell cycle as they initiate and maintain the phosphorylated state of the retinoblastoma protein (pRb), which plays a central role in proliferation. Cyclin B-CDK1 complex needs to be activated during the G2/M phase of the cell cycle to allow its progression (Lapenna and Giordano, 2009; Elbaz et al., 2012).

We also observed that p53 was downregulated at all concentrations tested. This tumor suppressor controls multiple cell cycle checkpoints regulating the mammalian response to DNA damage. Nakayama and Yamaguchi (Nakayama and Yamaguchi, 2013) suggested that the reduction of cyclin B1 levels may lead to the restoration of the p53 pathway, but not when A549 cells were treated with GEV. Also, cells died independently of cyclin B1 and p53 status. These results agree with the findings of Wang et al. (Wang et al., 2009) showing a downregulation of p53 by CGs digoxin and ouabain in multiple human cancer cell lines, independently of their p53 status. Moreover, these effects occurred preferentially in cancerous cells vs. non-cancerous cells, a differential effect also observed for GEV.

In conclusion, GEV reduced the proliferation and the viability of various cell types. In A549 cells, GEV triggered effects independent of, calpain, cathepsin, RIP-1 kinase, and PARP-1 inhibitors. Nevertheless, GEV triggered a non-canonical mode of cell death as caspase-mediated PS exposure was identified in selected sub-populations. Moreover, GEV downregulated cyclin B1 and p53 expression levels and caused an accumulation of cells in G2/M. In a leukemia model, GEV led to caspase-dependent apoptosis concomitant with an accumulation of cells in subG0. Overall, our results show the cytotoxic effects of GEV on A549 and U937 cells by inducing non-canonical cell death in solid tumor cells and canonical cell death in human leukemia. The solid tumor A549 cells exhibit a very high basal autophagic flux, compatible with their high proliferation rate. GEV does not further exacerbate autophagy, rather this compound seems may have some potentials to impair it, for example looking at the reduced accumulation of LC3I-II at earlier times of treatment and lover concentrations, a condition which was not prevented by bafilomycin A1. Inhibition of autophagy exacerbates cell death induction as a critical mechanism for cellular energy and nutrition homeostasis, as well as cell survival in many forms of cancer. As such, it could be interesting in the future to verify if GEV might be especially promising against autophagy-addicted cancer types characterized by improved stress and chemotherapy resistance.

## Ethics statement

This study was carried out in accordance and with the agreement of the recommendations of the SNU College of Pharmacy animal welfare legislation.

## Author contributions

Participated in research design: NS, ClC, WK, CS, and MarcD; Conducted experiments: NS, ClC, J-YL, AM, KK, AdC, and JM; Contributed new reagents or analytic tools: RP, WK, K-WK, ChC, KK, H-JK, BH, and FB; Performed data analysis: NS, ClC, ChC, KK, H-JK, BH, and MarcD; Wrote or contributed writing of the manuscript: NS, ClC, MarioD, CS, and MarcD.

### Conflict of interest statement

The authors declare that the research was conducted in the absence of any commercial or financial relationships that could be construed as a potential conflict of interest.

## Abbreviations

3-ABA: 3-aminobenzamide
BAF: bafilomycin A1
DMSO: dimethyl sulfoxide
FBS: fetal bovine serum
FCS: fetal calf serum
GEV: glucoevatromonoside
HT-29: human colon adenocarcinoma cells
Jurkat: T-cell leukemia
K562: human chronic myelogenous leukemia
LC3: microtubule-associated protein 1A/1B light-chain
MRC-5: normal lung fibroblast cell line
NEC: necrostatin
NSCLC: non small lung cancer cells
PARP: poly(ADP-ribose)polymerase
PBMCs: peripheral blood mononucleated cells
Raji: Burkitt's Lymphoma
RIPK-1: receptor-interacting protein kinase-1
SK-N-AS: human neuroblastoma cells
SH-SY5Y: human neuroblastoma cells
U937: histiocytic lymphoma.
