# Supplementary material for: Cardiac Glycoside Glucoevatromonoside Induces Cancer Type-Specific Cell Death
Source: Front Pharmacol. 2018 Mar 1;9:70. doi: 10.3389/fphar.2018.00070 (PMC5838923; doi:10.3389/fphar.2018.00070)
Supplement: Supplementary file 1 [file Table1.DOCX]

**Supplementary Table 1.** Effect of selected cardenolides on growth of A549 lung adenocarcinoma cells, compared to paclitaxel (positive control). Values represent the mean ± standard deviation (SD) of three independent experiments; Inhibitory concentration (IC)_50_ = concentration that inhibited 50% of A549 cell growth; Origin: (P) plant; (S) synthetic; (FB) biotransformation by fungi.

| **Compound Number** | **Cardenolide** | **Chemical structure** | **IC_50_ ± SD (nM)** | **Origin**  **[Reference]** |
| --- | --- | --- | --- | --- |
| **1** | Digitoxigenin |  | 62.7±5.1 | (P) [1] |
| **2** | Digitoxigenin monodigitoxoside |  | 33.5±2.6 | (P) [2] |
| **3** | Digitoxigenin bisdigitoxoside |  | 58.5±4.7 | (P) [2] |
| **4** | Glucoevatromonoside |  | 19.3±4.5 | (P) [2] |
| **5** | Glicodigifucoside |  | 63.1±13.3 | (P) [2] |
| **6** | Digitoxin |  | 368.5±21.8 | (P) [3] |
| **7** | β-acetyldigitoxin |  | 220.5±30.5 | (P) [2] |
| **9** | Gitoxigenin |  | >1000 | (P) [4] |
| **10** | Glicogitoroside |  | 609.7±28.0 | (P) [3] |
| **11** | Gitoxin |  | >1000 | (P) [4] |
| **12** | Digoxigenin bisdigitoxoside |  | 153.6±20.0 | (P) Extrasynthèse S.A., Genay, France |
| **13** | Digoxigenin tetradigitoxoside |  | 241.5±80.5 | (P) Extrasynthèse S.A., Genay, France |
| **14** | Lanatoside C |  | 264.6±18.4 | (P) [3] |
| **15** | Desacetyl –  lanatoside C |  | 133.8±50.9 | (P) [5] |
| **16** | β-methyl digoxin |  | 207.8±20.2 | (S) [6] |
| **18** | Digitoxin (peracetylated) |  | >1000 | (S) [7] |
| **19** | β-methyldigitoxin (peracetylated) |  | >1000 | (S) [7] |
| **20** | *Epi*-digitoxigenin  3α-OH |  | >1000 | (S) [1] |
| **22** | 21-*O*-malonyl-deoxycorticosterone |  | >1000 | (S) [8, 9] |
| **23** | 20,21-cetol of 3β-O-acetyl-digitoxigenin |  | >1000 | (S) [8, 9] |
| **24** | 3β-O-acetyl-digitoxigenin |  | >1000 | (S) [10] |
| **25** | 7β-hydroxydigitoxigenin |  | >1000 | (FB) [11] |
| **26** | 8β- hydroxydigitoxigenin |  | >1000 | (FB) [11] |
| **27** | Δ^14-15^digitoxigenin |  | >1000 | (S) [12] |
| **28** | Digoxigenin |  | >1000 | (P) [12, 13] |
| **29** | Digoxin |  | 133.8±7.0 | (P) Sigma-Aldrich (St. Louis, USA) |
| **30** | - |  | >1000 | (S) [12] |
| **31** | 14,15 β-Epoxy-14-anhydro-digoxin |  | >1000 | (S) [12] |
| **32** | - |  | >1000 | (S) [10] |
| **33** | - |  | >1000 | (S) [14] |
| **34** | - |  | >1000 | (S) [15] |
| **35** | - |  | >1000 | (S) [16] |
| **36** | - |  | >1000 | (S) [15] |
| **38** | K-strophantoside |  | 607.5±10.0 | (S) Carl Roth GmbH&Co. KG, Karlsruhe, German |
| **41** | 3β-[2-(1-amantadin)-1-on-ethylamine] digitoxigenin |  | 88.4±5.4 | (S) [17] |
| **42** | (3β,5β)-card-20(22)-enolide, 14-hydroxy-3-[(4-morpholinyl acetyl)amino] |  | 236.8±84.5 | (S) [18] |
| **43** | - |  | >1000 | (S) [19] |
| **45** | - |  | >1000 | (S) [14] |
| **47** | - |  | >1000 | (S) [20] |
| **55** | - |  | >1000 | (S) [10] |
| **57** | - |  | >1000 | (S) [16] |
| **59** | 3-(phosphite-O-dimethyl)-digitoxigenin |  | 795.3±14.4 | (S) [21] |
| **60** | 3-(phosphate-O-dimethyl)-digitoxigenin |  | 276.9±30.6 | (S) [21] |
| **65** | β-methyldigitoxin |  | 185.7±3.0 | (S) [22] |
| **66** | Convallatoxin |  | 32.8±1.9 | (P) Sigma-Aldrich (St. Louis, USA) |
| **68** | Cymarin |  | 123.2±3.9 | (P) [23] |
| **-** | Paclitaxel | - | 260.5±70.8 | (P) Sigma-Aldrich (St. Louis, USA) |

**Supplementary Table references**

[1] U. Stache, W. Fritsch, W. Haede, K. Radscheit, K. Fachinger, Herstellung von ungesättigten Lactonen der Steroid-Reihe, IV1) Synthese von Uzarigenin, Justus Liebigs Ann. Chem. 726 (1) (1969) 136-144.

[2] F. Castro Braga, W. Kreis, A. Braga de Oliveira, Isolation of cardenolides from a Brazilian cultivar of Digitalis lanata by rotation locular counter-current chromatography, J. Chromatogr. 756 (1-2) (1996) 287-291.

[3] F.C. Braga, W. Kreis, R.A. Recio, A.B. deOliveira, Variation of cardenolides with growth in a Digitalis lanata Brazilian cultivar, Phytochemistry 45 (3) (1997) 473-476.

[4] Y. Terada, R. Misoi, N. Watanabe, M. Hornberger, W. Kreis, Structure-specificity relationship of cardiac glycosides as a substrate for glucohydrolase II, Chem. Pharm. Bull. (Tokyo) 48 (3) (2000) 349-352.

[5] W. Kreis, E. Reinhard, Two-stage cultivation of Digitalis lanata cells: semicontinuous production of deacetyllanatoside C in 20-litre airlift bioreactors, J. Biotechnol. 16 (1-2) (1990) 123-135.

[6] E. Reinhard, W. Kreis, U. Barthlen, U. Helmbold, Semicontinuous cultivation of Digitalis lanata cells: production of beta-methyldigoxin in a 300-L airlift bioreactor, Biotechnol. Bioeng. 34 (4) (1989) 502-508.

[7] R.M.d. Pádua, Butenolide ring closure via a malonic ester of 21-hydroxy-pregnane, substrate synthesis, uptake, biotransformation and ring closure in vitro, Friedrich-Alexander-Universität Erlangen-Nürnberg (FAU), Naturwissenschaftliche Fakultät (Doctoral Thesis) (2009) urn:nbn:de:bvb:29-opus-13108.

[8] R.M. Padua, R. Waibel, S.P. Kuate, P.K. Schebitz, S. Hahn, P. Gmeiner, et al., A simple chemical method for synthesizing malonyl hemiesters of 21-hydroxypregnanes, potential intermediates in cardenolide biosynthesis, Steroids 73 (4) (2008) 458-465.

[9] U. Stache, K. Radscheit, W. Fritsch, W. Haede, H. Kohl, H. Ruschig, Herstellung von ungesättigten Lactonen der Steroidreihe, VII. Synthese von 4(5)-Dehydro-bufadienoliden, Justus Liebigs Ann. Chem. 750 (1) (1971) 149-164.

[10] T.W. Güntert, H.H.A. Linde, M.S. Ragab, S. Spengel, Synthese von 4-[3β, 14-Dihydroxy-5β, 14β-androstan-17β-yl]-3-pyrrolin-2-on (Hothesimogenin). Partialsynthetische Versuche in der Reihe der Herzgifte, 11. Mitteilung, Helv. Chim. Acta 61 (3) (1978) 977-983.

[11] R.M. Pádua, A.B. Oliveira, J.D. Souza Filho, J.A. Takahashi, M.d.A.e. Silva, F.C. Braga, Biotransformation of digitoxigenin by Cochliobolus lunatus, J. Braz. Chem. Soc. 18 (2007) 1303-1310.

[12] L. Sawlewicz, H.H. Linde, K. Meyer, [Epoxides of cardenolides and cardenolide glycosides, partial synthesis experiments in the seriers of the cardiotoxins], Helv. Chim. Acta 51 (6) (1968) 1353-1362.

[13] R.M. Pádua, A.B. Oliveira, J.D. Souza Filho, G.J. Vieira, J.A. Takahashi, F.C. Braga, Biotransformation of digitoxigenin by Fusarium ciliatum, J. Braz. Chem. Soc. 16 (2005) 614-619.

[14] T.W. Guntert, H.H. Linde, M.S. Ragab, S. Spengel, [Synthesis of a homologous 3beta-hydroxycard-5,22-dienolide (author's transl)], Helv. Chim. Acta 59 (6) (1976) 2125-2137.

[15] L. Sawlewicz, E. Weiss, H.H. Linde, K. Meyer, [245. 3-Alpha- and 3-beta-amino-3-deoxydigitoxigenin. Partial synthesis experiments in the series of heart poisons. 5], Helv. Chim. Acta 55 (7) (1972) 2452-2460.

[16] E. Hauser, U. Boffo, L. Meister, L. Sawlewicz, H.H. Linde, K. Meyer, [3-amino-3-desoxy compounds from uzarigenin, oleandrigenin, gitoxigenin, and digoxigenin. Partial synthesis experiments in the series of cardiac poisons. 6], Helv. Chim. Acta 56 (8) (1973) 2782-2795.

[17] E. Nolte, A. Sobel, S. Wach, H. Hertlein, N. Ebert, F. Muller-Uri, et al., The New Semisynthetic Cardenolide Analog 3beta-[2-(1-Amantadine)-1-on-ethylamine]-digitoxigenin (AMANTADIG) Efficiently Suppresses Cell Growth in Human Leukemia and Urological Tumor Cell Lines, Anticancer Res. 35 (10) (2015) 5271-5275.

[18] X. Qian, Certain chemical entities, compositions, and methods, Neupharma (2016) United States Patent 20160039865.

[19] G.R. Lenz, J.A. Schulz, Formation of 14-Alpha-Cardenolides from 21-Acetoxy-20-Keto Steroids, J. Org. Chem. 43 (12) (1978) 2334-2339.

[20] S. El-Dine, K. Faust, T.W. Güntert, E. Hauser, H.H.A. Linde, S. Spengel, Die Synthese von 3β-Acetoxy-24-aza-24-desoxa-xysmalogenin,. Partialsynthetische Versuche in der Reihe der Herzgifte. 12. Mitteilung, Helv. Chim. Acta 62 (4) (1979) 1283-1287.

[21] L. Sawlewicz, H.H. Linde, K. Meyer, [3-O-phosphoryl-digitoxigenin], Helv. Chim. Acta 53 (6) (1970) 1382-1385.

[22] M. Heins, J. Wahl, H. Lerch, F. Kaiser, E. Reinhard, Preparation of beta-methyldigoxin by hydroxylation of beta-methyldigitoxin in fermenter cultures of Digitalis ianata, Planta Med. 33 (1) (1978) 57-62.

[23] K.H. Segel, Iritierte Verbindungen. I. Cymarol-(19-3H) (3beta, 5beta, 14beta, 19-Tetraoxy-cardenol-(19-3H)-D-cymarosid), Journal fuer Praktische Chemie 13 (3-4) (1961) 152-156.
