# Supplementary material for: Cardiac Glycoside Glucoevatromonoside Induces Cancer Type-Specific Cell Death
Source: Front Pharmacol. 2018 Mar 1;9:70. doi: 10.3389/fphar.2018.00070 (PMC5838923; doi:10.3389/fphar.2018.00070)
Supplement: Supplementary file 13 [file Presentation1.pdf]

Supplementary Figure Legends

Supplementary Figure 1. Effects of glucoevatromonoside on PBMC subpopulations. Analysis of monocytes (a) and lymphocytes (b) isolated from human blood of healthy donors and incubated with different concentrations of GEV (0-1000 nM). Cells were incubated for 24 and 48 h. Data represent the mean ± SD of five independent experiments; $*p < 0.05$ (ANOVA followed by Dunnett's test) when compared to untreated controls.

Supplementary Figure 2. Effects 10 and 50nM Glucoevatromonoside on LC3I/II conversion in A549. (a)Western blot analysis of LC3-II protein levels in A549 cells treated with GEV (50 nM) and co-treated with bafilomycin after 6, 12 and 18 h and (b) GEV (10 nM) in presence or absence of bafilomycin after 12 and 24 h. PP2 was used as positive control. β-actin was used as loading control. The blots shown are representative of three independent experiments. Expression levels of proteins were quantified by using ImageJ software (National Institutes of Health, Bethesda, USA). Numbers below western blot signal represent quantification of LC3II/LC3-I proteins ratio using β -actin as control for sample input.

Supplementary Figure 3. Effects of different inhibitors of cell death on A549 cells treated with glucoevatromonoside. Representative bright filter images of treated cells taken each three hours obtained with IncuCyte^{TM} videomicroscopy of cells treated with GEV (50 nM) and in presence of modulators; graphs represent the percentage of proliferation up to 72 h. (a) 3-aminobenzamide (PARPi), (b) RIP necrostatin, (c) bafilomycin A1 and (d) calpain (PD 150606). The data represent the mean ± SD of three independent experiments; $*p < 0.05$ (ANOVA followed by Dunnett's test) when compared to untreated controls.

Supplementary Figure 4. Absence of modulatory effect of inhibitors of cathepsines B, D and L on glucoevatromonoside-induced cell death in A549.
(a) Representative bright filter images of cells treated with GEV in presence or absence of Cathepsines B, D, and L by IncuCyte^{TM} and (b) Percentage of Ann+Pi-, Ann+PI+ and Ann-PI- A549 cells after 48h of GEV treatment in presence or absence of Cathepsines B, D, and L (two independent experiments).

Supplementary Figure 5. Glucoevatromonoside (GEV) does not produce the same alterations on U937 cells cycle as observed for A549 cells. Effects of GEV on U937 cells cycle distribution after 8, 16, 24 and 48 h of treatment at 10, 50 and 100 nM. The values represent the percentages of U937 cells in the indicated phases of cell cycle (subG0, G0/G1, S, and G2/M). The data represent the mean ± SD of three independent experiments; $*p < 0.05$, $**p < 0.01$ and $***p < 0.001$ (ANOVA followed by Dunnett's test) when compared to untreated controls.

Supplementary Videos

Supplementary Video 1. Representative full-length 72 h brightness contrast images were taken each three hours on an IncuCyte^{TM} of A549 untreated cells for 72 hours.

Supplementary Video 2. Representative full-length 72 h brightness contrast images were taken each three hours on an IncuCyte^{TM} following exposure treatment of A549 cells to GEV (50 nM) for 72 hours.

Supplementary Video 3. Representative full-length 72 h brightness contrast images were taken each three hours on an IncuCyte^{TM} following exposure treatment of A549 cells to etoposide VP16 (50µM) for 72 hours.

Supplementary Video 4. Representative full-length 72 h brightness contrast images were taken each three hours on an IncuCyte^{TM} following exposure treatment of A549 cells to zVAD (50µM) for 72 hours.

Supplementary Video 5. Representative full-length 72 h brightness contrast images were taken each three hours on an IncuCyte^{TM} following exposure treatment of A549 cells to GEV (50 nM) + zVAD (50µM) for 72 hours.

Supplementary Video 6. Representative full-length 72 h brightness contrast images were taken each three hours on an IncuCyte^{TM} following exposure treatment of A549 cells to PARPi (5 mM) for 72 hours.

Supplementary Video 7. Representative full length 72 h brightness contrast images were taken each three hours on an incuCyte^{TM} following exposure treatment of A549 cells to GEV (50 nM) + PARPi (5mM) for 72 hours.

Supplementary Video 7. Representative full-length 72 h brightness contrast images were taken each three hours on an IncuCyte^{TM} following exposure treatment of A549 cells to NEC-1 (160 µM) for 72 hours.

Supplementary Video 8. Representative full-length 72 h brightness contrast images were taken each three hours on an IncuCyte^{TM} following exposure treatment of A549 cells to GEV (50 nM)+ NEC-1 (160µM) for 72 hours.

Supplementary Video 9. Representative full-length 72 h bright-field images were taken each three hours on an incuCyte^{TM} following exposure treatment of A549 cells to BAF (10 nM) for 72 hours.

Supplementary Video 10. Representative full-length 72 h bright-field images were taken each three hours on an incuCyte^{TM} following exposure treatment of A549 cells to GEV (50 nM) + BAF (10 nM) for 72 hours.

Supplementary Video 11. Representative full-length 72 h bright-field images were taken each three hours on an incuCyte^{TM} following exposure treatment of A549 cells to calpain inhibitor PD 150606 (50µM) for 72 hours.

Supplementary Video 12. Representative full-length 72 h bright-field images were taken each three hours on an incuCyte^{TM} following exposure treatment of A549 cells to GEV (50 nM) + calpain inhibitor PD 150606 (50µM) for 72 hours.

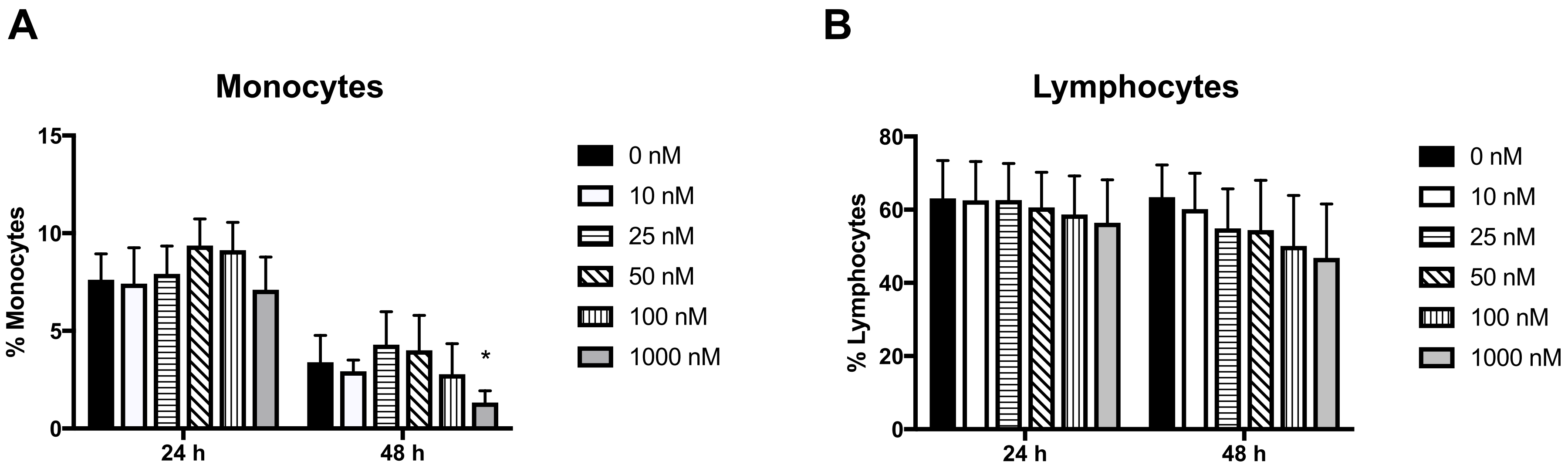

Supplementary Figure 1

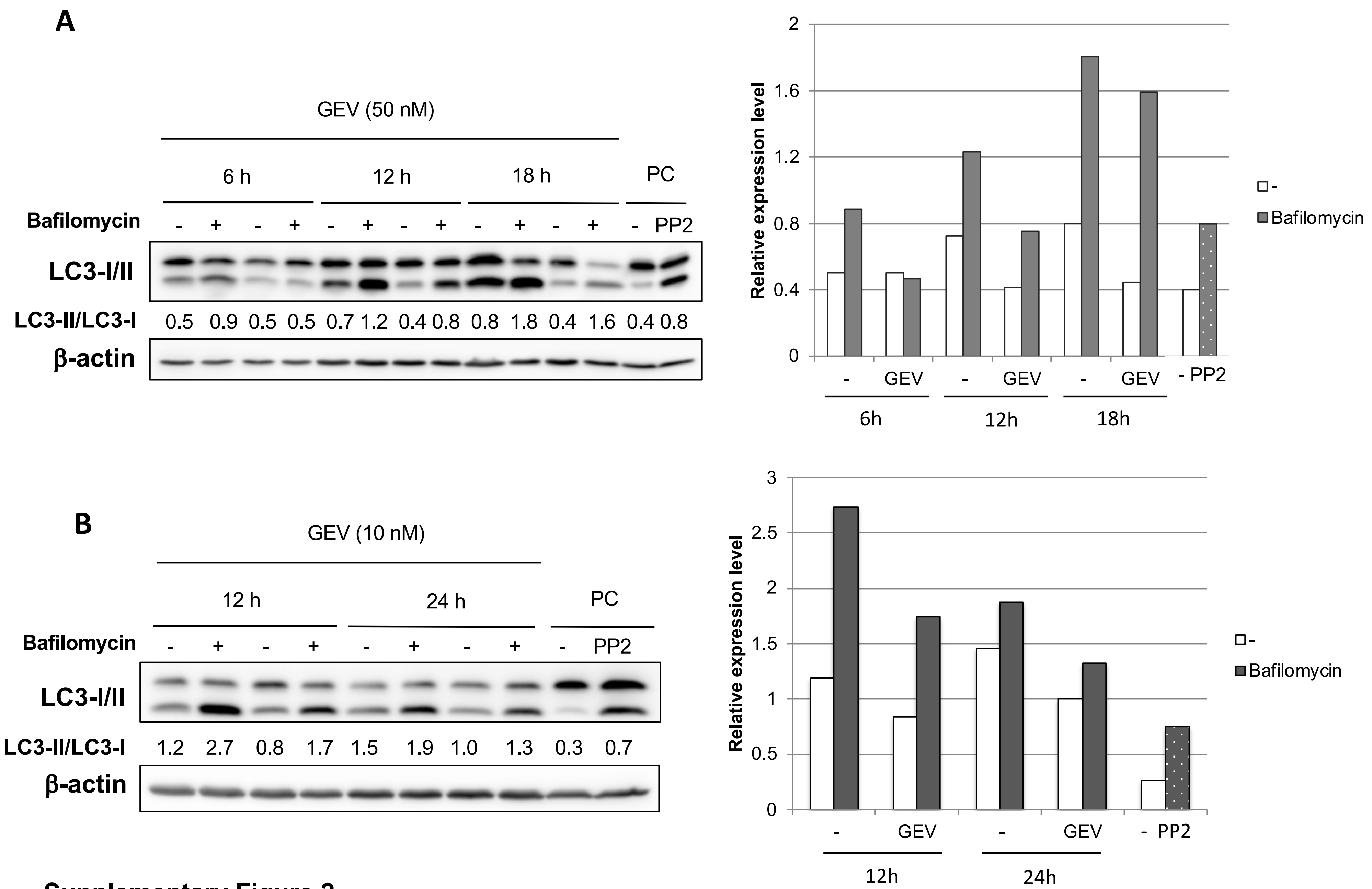

Supplementary Figure 2

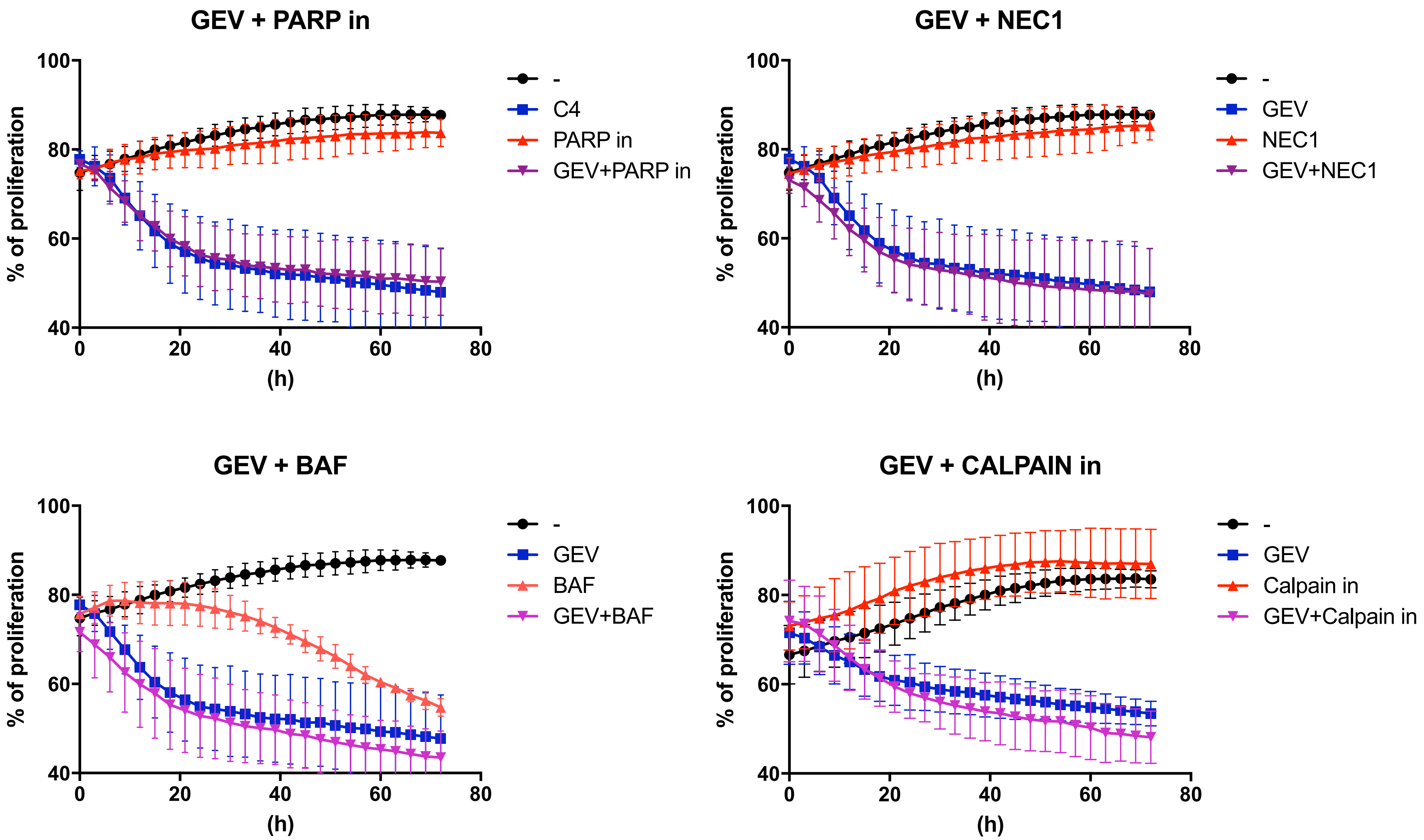

Supplementary Figure 3

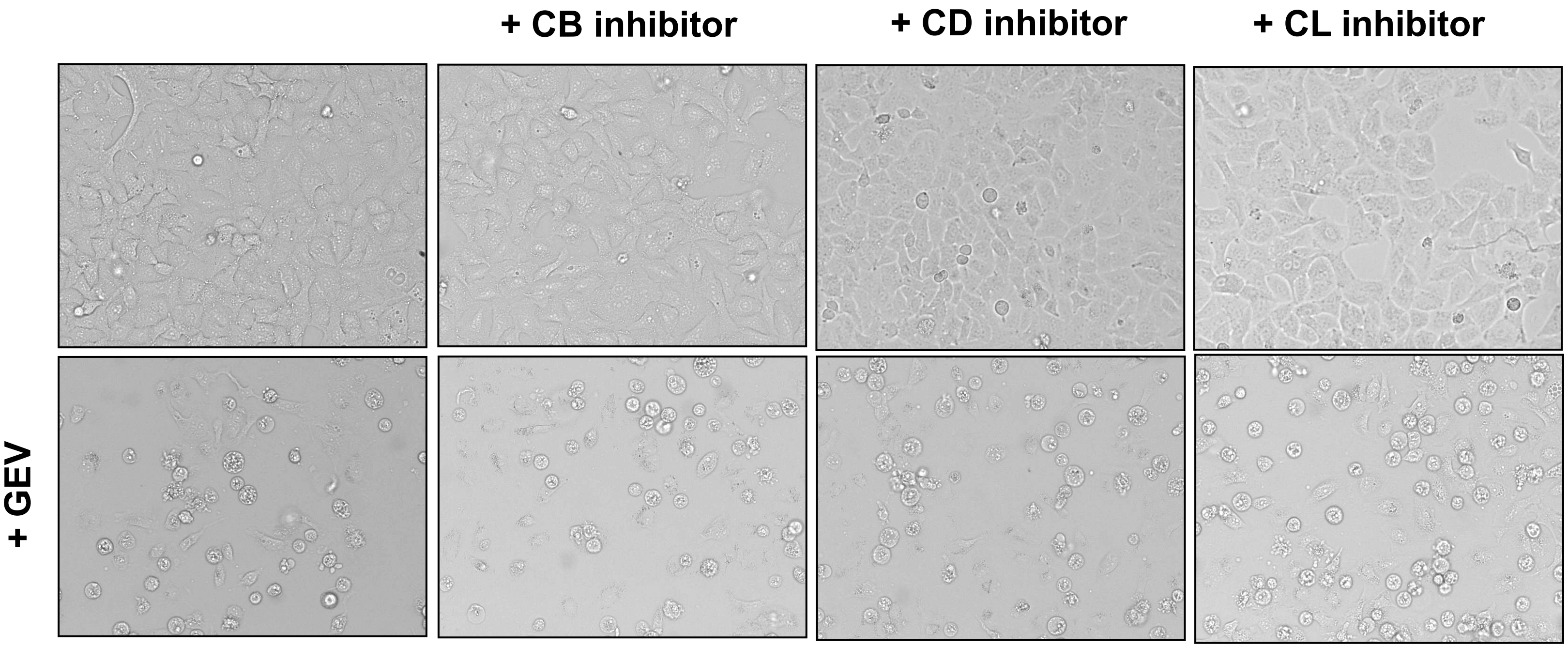

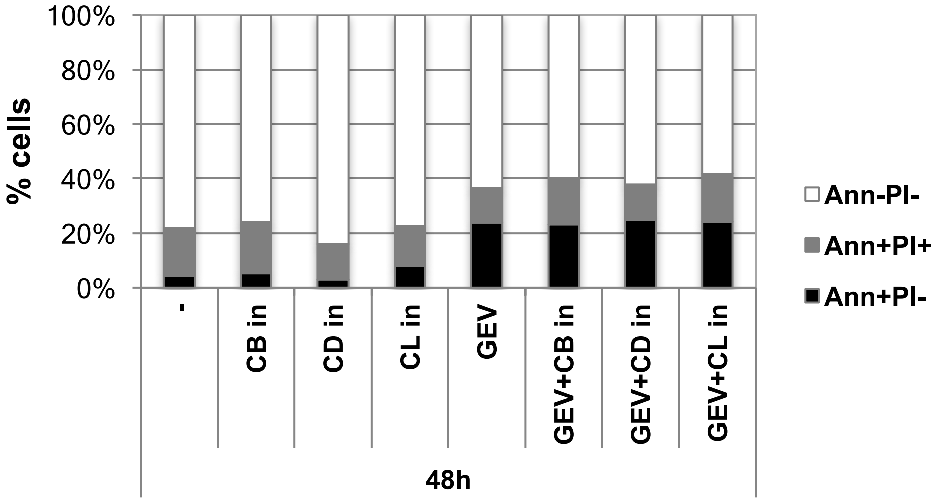

Supplementary Figure 4

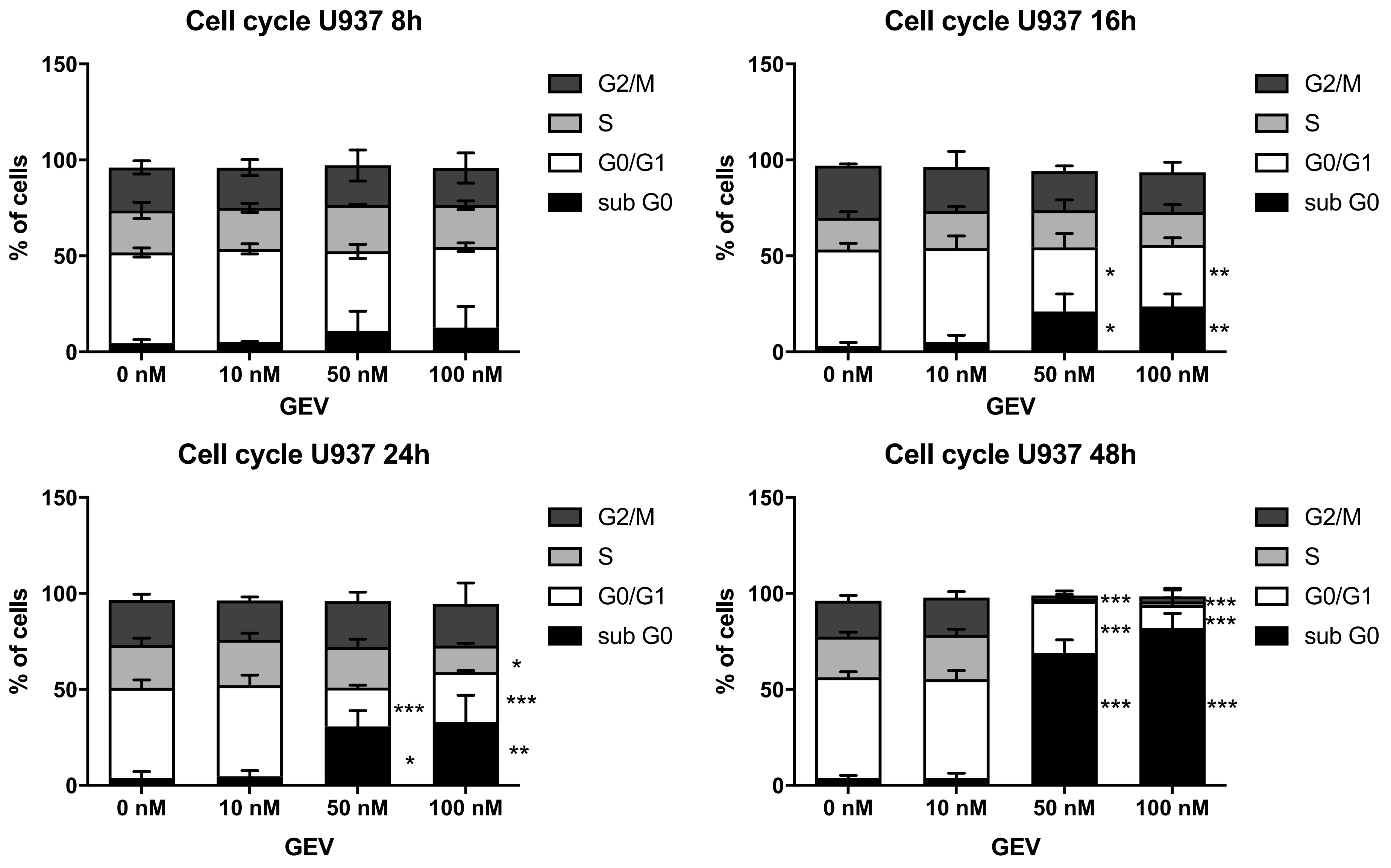

Supplementary Figure 5
